# A Simple and Efficient One-Step Synthesis System for Flexible Production of Circular RNA in *E. coli*

**DOI:** 10.3390/biom14111416

**Published:** 2024-11-07

**Authors:** Xiayang Zhao, Yiqing Liu, Huanhui Huang, Yue Sun, Fangli Wu, Weibo Jin

**Affiliations:** 1 Key Laboratory of Plant Secondary Metabolism and Regulation of Zhejiang Province, College of Life Sciences and Medicine, Zhejiang Sci-Tech University, Hangzhou 310018, China; 202120801102@mails.zstu.edu.cn (X.Z.); 2023210901042@mails.zstu.edu.cn (Y.L.); 202220901020@mails.zstu.edu.cn (H.H.); wfl@zstu.edu.cn (F.W.); 2 Zhejiang Sci-Tech University Shaoxing Academy of Biomedicine, Zhejiang Sci-Tech University, Shaoxing 312366, China; 2401130109@nbu.edu.cn

**Keywords:** group II intron, circularization in vivo, one-step synthesis, sciRNA, condition optimization

## Abstract

Circular RNA (circRNA) exhibits a higher stability and intracellular half-life than linear RNA and has better potential in the fields of RNA vaccines and RNAi drugs. The current strategies for circRNA preparation have low efficiency, high costs, and high complexity, which significantly limits their applications. In this paper, we propose a one-step synthesis of circRNA based on *E. coli.* The four RNA sequence lengths of 1700, 1400, 500, and 64 nt were connected to group II intron elements from the surface protein region of *Clostridium tetani* and then inserted downstream of the T7 promoter in the pET28a plasmid to assist in cyclization. Then, circRNA was produced in HT115, where the yields of pET28-1700, pET28-1400, pET28-500, and pET28-64 were improved to 820, 783, 691, and 460 ng/1 mL, respectively. Consequently, this system could achieve the mass production of circRNA using only a simple *E. coli* culture and inducible expression. Meanwhile, the overexpressed circRNA and small circular interference RNA (sciRNA) maintained their biological functions in the protein translation and RNAi. Therefore, this simple and efficient one-step synthesis system can be applied to the functional study and preparation of circRNA in the future.

## 1. Introduction

Circular RNA (circRNA) is a covalently closed non-coding RNA molecule that is expressed at low levels in organisms and produced by the back-splicing of pre-mRNA in eukaryotes [1]. CircRNAs play many important roles, such as regulating transcription and acting as miRNA sponges [2], and a few circRNAs can even encode peptides. Due to their unique structures, circRNAs have better endonuclease resistance than traditional linear RNA. CircRNA has shown great potential in circRNA vaccines and gene therapy. Studies have shown that circRNA, when used in an RNA vaccine, is less susceptible to degradation by nucleases, has higher stability, and has lower immunogenicity [3]. Additionally, circRNA vaccines achieve a longer half-life and higher expression efficiency than linear mRNA vaccines [4,5]. Furthermore, with their ring structures, circRNAs provide an alternative method to enhance the stability of siRNA, which leads to a longer interference effect for their effects as an RNAi drug [6,7]. In short, the demand for circRNA in the medical field has been increasing in recent years; therefore, a large-scale, low-cost, and sustainable production strategy for circRNA is urgently needed.

The current main in vitro synthesis method for circRNA is to connect the end of the linear RNA precursor to a covalent closed loop using, e.g., chemical ligation, enzymatic ligation, or ribozyme methods. Bromo acetonitrile and 1-ethyl-3-(3′-dimethylaminopropyl) carbodiimide are the reagents used to connect DNA-RNA hybrids and synthesize circRNA, but they have a low circularization efficiency and present significant safety concerns [8]. T4 DNA ligase (T4 Dnl) [9], T4 RNA ligase 1 (T4 Rnl 1) [10], and T4 RNA ligase 2 (T4 Rnl 2) [11] have been used to circularize circRNAs, showing a higher efficiency and fewer safety concerns. However, these methods still require adjuvant involvement and are cumbersome and costly. Ribozymes have been used to achieve the efficient circularization of RNA precursors using its splicing properties. The modified group I intron self-splicing system, which is also known as the permutation of intron exon (PIE) method, is the most used enzyme connection method, and can be combined with Mg^2+^ and GTP to connect flanking exon sequences. This method synthesizes circRNAs ranging from 1 to 5 kb in length, but these circRNAs include an additional 74 to 186 nt of nucleotides, respectively [12,13]. The folded conformation of circRNA may be altered to trigger an immune response because these sequences pair with the internal sequence of the circRNA or the foreign sequence itself forms a stable double-stranded structure [2]. Furthermore, this method for circularizing short fragments is not satisfactory in vitro since most group I introns are not accurately spliced. Other ribozymes derived from subviral entities (satellite viruses and the hepatitis delta virus), such as hairpin ribozymes (HPRs) and hammerhead ribozymes, have also been applied to circRNA synthesis. For example, HPRs have been used to produce circRNA from circular single-stranded DNA templates [14]. However, this strategy retains the ribozyme activity, which leads to conformational instability and potential off-target effects. Although there have been extensive studies and applications of in vitro circRNA synthesis, this strategy still faces challenges, such as high production and purification costs, cumbersome steps, and the introduction of non-essential nucleotide residues. Therefore, novel strategies for in vivo circRNA synthesis have gained widespread attention.

Compared with in vitro synthesis, the in vivo synthesis of circRNA has the advantages of simplicity and a higher synthesis efficiency. In a previous study, ALU introns [15] were used to efficiently circularize 51–668 nt RNAs in vivo. However, this method required the target sequence to pair with the intron scaffold, and competitive splicing reactions in vivo led to additional byproducts. The flanking intron of the Drosophila MBL gene was used to circularize RNA in cells, but the overexpression of the MBL protein was essential during the synthesis of circRNA [16]. A system for the efficient and large-scale production of circRNA that ensures low cytotoxicity was produced using Twister ribozymes [17]. Moreover, circRNAs were produced in *E. coli* using the group I intron [18,19]; this method also provides a strategy for the in vivo production of circular small RNA [20]. However, using group I introns for circRNA synthesis also introduces nonessential exogenous sequences.

Group II introns are a class of ribozymes composed of intron RNA and intron-encoded proteins that have self-splicing capabilities. A previous study constructed the PIE method for the in vitro generation of circRNA sequences using group II introns arranged in the yeast mitochondrial genome [21]. Unlike the splicing mechanism of group I introns, group II introns produce circRNA that does not require the participation of the natural exon sequence. All group II introns have six domains, and the splicing specificity is determined by several short exon binding sites (EBSs) in domain 1. The self-splicing of group II introns occurs in vitro with the correct folding of the intron RNA and the presence of Mg^2+^. Based on their overall structural similarities, group II introns are divided into three families: IIA, IIB, and IIC [22]. However, the splicing site recognition sequences at the 5′ and 3′ ends are different between these families. IIA and IIB determine the 5′ splicing position by splicing EBS1 and EBS2 with intron binding site 1 (IBS1) and IBS2 at the 5′ end, while IIC only uses EBS1 to recognize the 5′ splicing position. For the recognition of the 3′ end splicing position, IIA pairs the δ base upstream of the EBS1 with the first nucleotide (δ’) of the exon sequence at the 3′ end, while the IIB and IIC introns pair EBS3 with IBS3 [23]. S. Mikheeva et al. [21] first demonstrated the precise splicing of a yeast group II intron in vitro to splice the first kringle domain (K1) of human tissue and complete the RNA circularization. In 2022, Chuyun Chen et al. [24] developed a self-catalytic system based on the *Clostridium tetani* (ctSLP) group II intron, which efficiently and scarlessly produced >1300 nt circRNAs with a rational sequence design that functions in translation both in vitro and in vivo. Compared with other circularization methods, group II introns have precise splicing, have a wide-ranging sequence adaptability, and can complete circularization reactions without introducing any exogenous sequences. This provides a new strategy for the efficient and precise production of circRNA. However, currently, the synthesis of circRNA using group II introns is generally achieved through in vitro synthesis. The disadvantage of unsustainable production still limits circRNA production using this method, and the feasibility of <1000 nt circRNA production has not been verified.

In this study, we used group II introns for circRNA preparation in *E. coli*. Moreover, we explored the circularization efficiency for four lengths of RNA fragments, where the shortest one was only 64 nt. The circRNA produced using this method showed a similar protein expression and RNAi ability to circRNA produced in vitro. By optimizing this system, we developed a large-scale, low-cost, and sustainable preparation of circular RNA for protein expression and RNAi based on the self-splicing mechanism of group II introns.

## 2. Materials and Methods

### 2.1. Vector Construction

The active ribozyme was a group II intron from *C.te.I1*, which was located in the surface protein region of *C.te.I1*. The sequences of the *C.te.I1* ribozyme with a six-domain sequence (D1, D2, D3, D4, D5, D6) were chemically synthesized as fragment 1 (D1, D2, D3, D4′) and fragment 2 (D4′, D5, D6) [25] from GENEWIZ (Guangzhou, China). Different lengths (1700, 1400, 500, 64 bp) of target fragments were chemically synthesized; these sequences were concatenated from GENEWIZ (see the Supplementary Data for the sequences of these segments).

The pET-28a (+) plasmid was used as the overexpression plasmid, where it was double-digested with XhoI (New England Biolabs, Ipswich, MA, USA) and BglII (New England Biolabs, USA) enzymes. Then, the D4′ end of fragment 1 was ligated to the XhoI site, while the D4′ end of fragment 2 was ligated to the BglII site via its overlapping with the PCR (see Table S1 for the primers), which resulted in the construction of a group II intron splicing cassette for sequence circularization.

### 2.2. In Vitro Transcription (IVT) and Circularization of RNA

The plasmid DNAs were linearized with XhoI digestion and purified with a DNA gel recovery kit (UElandy, Suzhou, China). The 200 ng linearized DNA was used as a template for in vitro transcription with the T7 RiboMAX™ Large-Scale RNA Production System (Promega, Madison, WI, USA) according to the manufacturer’s instructions. After the DNase I treatment, the RNA products were column-purified with an RNAclean Kit (TIANGEN, Beijing, China) to remove excess NTP and other salts in the IVT buffer, as well as the possible small RNA fragments generated during the IVT. The purified RNA was further circularized in a new circularization buffer [25]. The RNA was first heated to 90 °C for 1 min, and then cooled down to 75 °C for 5 min and cooled down to 53 °C for 15 min, after which a buffer including indicated magnesium and ammonium ion was added to a final concentration of 40 mM Tris-HCl at pH 7.5, 500 mM NH_4_Cl, and 100 mM MgCl_2_, and then incubated at 53 °C for 10 min for circularization. A total of 5 μL circular RNA of interest was assessed through 2% agarose gel electrophoresis at 80 V for 30 min.

### 2.3. Establishment and Optimization of In Vivo Circular RNA (circRNA) Production System

#### 2.3.1. Establishment of the Expression Strain System

As Figure S1 shows, to transform the recombinant plasmid into *E. coli* HT115 (DE3), positive colonies were cultured in a liquid LB medium that contained 0.1 mg/ml kanamycin at 37 °C and 220 rpm until the OD_600_ reached 0.6. Then, IPTG was added to a final concentration of 0.1 mg/mL, and the culture was induced at 37 °C and 220 rpm for 4 h. After the induction, the bacterial cells were collected using centrifugation at 7012 g for 1 min. The supernatant was discarded, and the cell pellet was slowly resuspended in a 1/10 volume of DEPC-treated water. An equal volume of phenol (Solarbio, Beijing, China) was added to the suspension, and the mixture was incubated at 64 °C in a water bath for 5 min. After the mixture was completely cooled on ice, an equal volume of chloroform (Gaojing Chemical, Hangzhou, China) was added, and the mixture was vigorously shaken for 1 min. It was then centrifuged at 15,777× *g* for 15 min, and the upper aqueous phase was collected. To this, an equal volume of chloroform was added, and the mixture was vigorously shaken for 1 min. Once again, the mixture was centrifuged at 15,777× *g* for 10 min, and the upper aqueous phase was collected, which was the crude extract of circular RNA.

A total of 500 μL RNA was transferred into a 2 mL tube, and 1.25 mL LiCl (7.5 M) was added. After mixing carefully, the mixture stood at 4 °C for 2 h. Then, it was centrifuged at 15,777× *g* for 15 min, and the supernatant was removed. Finally, 50 μL nuclease-free water was used to resolve the RNA. Subsequently, 5 μL of the expression of the circular RNA of interest was assessed through 2% agarose gel electrophoresis at 80 V for 30 min.

#### 2.3.2. Extraction Solution Exploration

According to the method described in Section 2.3.1, DEPC water was replaced with a Tris-Mg^2+^ extraction buffer without RNase (10 mmol/L Tris-HCl, pH 8; 10 mmol/L MgAc). The remaining steps of the method in Section 2.3.1 were followed and 5 μL of the expression of the circular RNA was analyzed using 2% agarose gel electrophoresis (80 V, 30 min).

#### 2.3.3. Exploration of Culture Medium Components

The LB culture medium was prepared with different final concentrations of Mg^2+^ (0, 10, 20, 50, 100, 150 mM) based on the splicing conditions of the group II introns (which involved only Mg^2+^). Culture-positive colonies were produced in the respective media and RNA extraction was induced using the method described in Section 2.3.2. The 5 μL expression of circular RNA was analyzed using 2% agarose gel electrophoresis (80 V, 30 min).

#### 2.3.4. Exploration of Induction Temperature

Culture-positive colonies were produced according to the method in Section 2.3.1 until the OD_600_ reached 0.6. IPTG was added at a final concentration of 0.1 mg/ml before different temperatures (23, 30, 37, 44 °C) were applied with agitation at 220 rpm for 4 h. The target RNA was extracted to analyze the 5 μL expression of the circRNA using 2% agarose gel electrophoresis (80 V, 30 min).

### 2.4. Validation of RNA Cyclization

#### 2.4.1. RNase R Cleavage Assay

The 1 μg of purified circRNA was digested using RNaseR (Beyotime, Shanghai, China) according to the manufacturer’s instructions. After the digestion, 2% agarose gel (80 V, 30 min) or 8% page gel (80 V, 80 min) was used for the analysis.

#### 2.4.2. Assay of circRNA with Reverse Transcription-Polymerase Chain Reaction (RT-PCR)

A pair of reverse PCR primers and a reverse transcription-specific primer were designed (Tables S2 and S3) for amplifying the breakpoint. Reverse transcription was performed using the PrimeScript™ RT Master Mix (Perfect Real Time) kit (TaKaRa, Shiga, Japan) according to the manufacturer’s instructions. Using the obtained cDNA as a template, reverse PCR was performed with primers (Table S3) that can amplify transcripts across the splice junction. These were then analyzed using a 2% agarose gel (120 V, 30 min).

#### 2.4.3. Sequencing of the PCR Products

The target fragment obtained from reverse PCR was purified using a DNA gel recovery kit (UElandy, Suzhou, China). Then, TA cloning of the purified product was performed using the 5 min TA/Blunt-Zero Cloning Kit (Novogene, Beijing, China) according to the manufacturer’s instructions. The TA cloning product was transformed into Top10 competent cells. The positive colonies were sequenced using Sanger sequencing to validate the back-splice junction of the circRNA.

### 2.5. Measurement of circRNA Translation and RNAi Effect

#### 2.5.1. Cytotoxic Activity of circRNA

Vero cells were cultured in 96-well plates, and then transfected with 1.5 and 3 μg circRNA produced in vitro and in vivo, respectively. After 1, 4, 7 days of transfection, the medium was removed and washed twice with PBS. Then, 90 μL of DMEM (Thermo Fisher Scientific, Waltham, MA, USA) medium with 10 μL of CCK8 (Solarbio, Beijing, China) was added to each well; each well was subsequently incubated for 3 h and recorded at 450 nm.

#### 2.5.2. GFP Fluorescence Identification

Human embryonic kidney cells (293 T) were cultured in a DMEM complete medium (Gibco, Jenks, OK, USA) that contained 10% fetal bovine serum (FBS, Gibco, USA) and 1% penicillin–streptomycin (Gibco, USA). When the cell confluency reached 70–80%, the cells were digested using trypsin (Gibco, USA), resuspended, and plated in a 12-well plate with 5 × 10^5^ cells per well. The plate was incubated at 37 °C and 10% CO_2_ for 12 h. The complete medium was removed and 1 mL of Opti-MEM™ I reduced-serum medium (Thermo Fisher Scientific, USA) was added to each well before incubation at 37 °C and 10% CO_2_ for 1 h. The cells were transfected with DEPC water, in vitro synthesized with cRNA-1400, and in vivo synthesized with cRNA-1400 using a Lipofectamine 2000 transfection reagent (Thermo Fisher Scientific, USA) at a ratio of 1:5 (Lipofectamine 2000/RNA). The cells were incubated at 37 °C and 10% CO_2_ and the fluorescence expression was analyzed at 6, 12, 24, and 48 h post-transfection.

#### 2.5.3. qPCR Analysis

Hep 3B cells were cultured in 6-well plates, then transfected with 1.5 μg siRNA and circRNA produced in vitro and in vivo, respectively. A total of 1, 3, 5, and 7 days after transfection, Hep 3B cells were harvested and total RNA was extracted used FastPure^®^ Cell/Tissue Total RNA Isolation Kit V2 (Vazyme, Nanjing, China). cDNAs were synthesized via reverse transcription using PrimeScript™ RT reagent Kit (Takara, Japan) according to the manufacturer’s instructions. The sequences of the primers are as follows: JNK-F: 5′-GAAACTAAGCCGTCCTTTTCA-3′ and JNK-R: 5′-GATCCAGCTCCATGTGAATAACC-3′. The reaction gradient was 42 °C for 15 min and 85 °C for 5 s. qPCR was performed with 2 × Phanta Flash Master Mix (Dye Plus) (Vazyme, China), and the data were analyzed using a Graph pad.

#### 2.5.4. Analysis of Apoptosis

Hep 3B cells were cultured in 6-well plates, then transfected with 1.5 μg siRNA and circRNA produced in vitro and in vivo, respectively. After three days of transfection, cells were harvested and treated using the Annexin V-FITC Apoptosis Detection Kit (Beyotime, Shanghai, China). Cells were analyzed using a BD Accuri C6 Plus (BD Biosciences, San Jose, CA, USA). Untransfected and undyed cells were used as a negative control for gating FITC and PI fluorescence. Single-dye cells were used as a positive control for gating FITC or PI fluorescence.

Plots were generated using FlowJo V10 software (Tree Star, Inc., Ashland, OR, USA).

### 2.6. Statistical Analysis

#### 2.6.1. Expression Analysis

The expression levels of circRNA obtained after in vivo induction with the optimized condition using 2% agarose gel (run at 80 V for 30 min) were compared and the gel images were analyzed using Image J 5.3; values are reported as mean ± s. e. m (*n* = 3).

The expression levels of *jnk* were evaluated using Graphpad Prism 5 (GraphPad Inc, San Diego, CA, USA). An ANOVA test was used to compare the differences among the four groups. A *p*-value of less than 0.05 was evaluated as statistically significant. Values were reported as mean ± standard deviation.

#### 2.6.2. Circularization Rate Analysis

The circRNA obtained after in vivo induction was digested by RNase R, and run in a 2% agarose gel (80 V, 30 min); the grayscale values before and after digestion were analyzed using Image J, and the circularization rate was calculated via Formula (1) before the circRNA production was calculated according to Formula (2); values are reported as mean ± s. e. m (n = 3).

(1) $$circularization\;rate\;(\%)=\frac{circRNA\;levels\;after\;RNase\;R\;digestion}{circRNA\;levels\;before\;RNase\;R\;digestion}\;*\;100\%$$

(2) $$circRNA\;production\;(\mathrm{ng}/1\;\mathrm{mL})=\frac{C\left(RNA\right)*V\left(RNA\right)*circularization\;rate}{V(E.coli)}$$

## 3. Results

### 3.1. Construction and In Vitro Transcription Validation of Four Different-Sized Circular RNA Expression Plasmids

**Figure 1:**
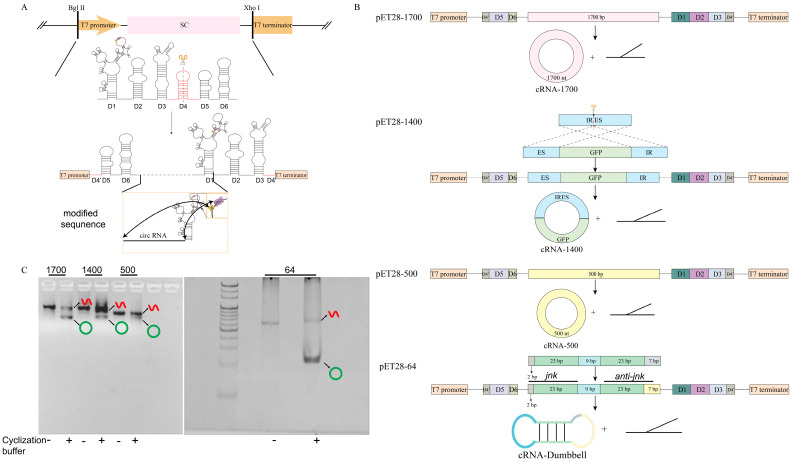
Construction and validation of expression plasmids for the synthesis of circular RNA using the Group II intron one-step method. (**A**) The expressions constructed by plasmids. The group II intron splicing sequence (D1, D2, D3, D4, D5, D6) was cleaved into fragment 1 (D4’, D5, D6) and fragment 2 (D1, D2, D3, D4’), and the circRNA sequence was inserted between D6 and D1. Then, the T7 promoter was ligated upstream of fragment 1 to form a group II intron splicing cassette (SC). Finally, the SC sequence was inserted between the Bgl II and Xhol restriction sites on the pET-28a plasmid and the circRNA expression plasmid was obtained. The modified sequence is the sequence of changes that need to be made according to the design sequence, including the circRNA sequence, EBS1, and the δ base of D1. (**B**) The sequence design of four circRNAs. Sequences of 1700 bp and 500 bp were inserted directly between D6 and D1. The IRES sequence was split into two fragments (IR and ES), and the GFP sequence was inserted between ES and IR to form a 1400 bp sequence. Loop 2 of the 64 bp sequence was split into loop2′ and loop2, and the sense strand, loop1 and antisense strand were inserted between loop2’ and loop2 in sequence. (**C**) cRNA−1700, cRNA−1400, cRNA−500, and cRNA−Dumbbell were cyclized in vitro; 1700, ‘group of cRNA−1700’; 1400, ‘group of cRNA−1400’; 500, ‘group of cRNA−500’; 64, ‘group of cRNA−Dumbbell’; -, ‘no circularization buffer added’; ‘+’, ‘circularization buffer added’; red linear symbol, ‘linear RNA’; green ring symbol, ‘circRNA’. Original images can be found in Supplementary Materials.

At present, group II introns are generally used for cyclization analyses of fragments larger than 1.3 kb [24]. To investigate the circularization efficiency of the group II introns for small RNA fragments, this study constructed several smaller fragment lengths and analyzed their circularization using the group II introns. The six domains of the group II intron were spliced and reconstituted into fragment 1 (D1, D2, D3, D4′) and fragment 2 (D4′, D5, D6) (Figure 1A). The coding DNA sequences of four circRNAs with sizes of 1700, 1400, 500, and 64 bp were inserted downstream of the group II intron D6 reported by Bonnie A McNeil [25]. Furthermore, the 3′ end of the sequence was complementary to the IBS1 of D1 (purple) and the 5′ end was complementary to the δ base of D1 (yellow, Figure 1A). The entire fragment was fused with a T7 promoter sequence at the 5′ end and inserted into the pET28a plasmid using the BglII and XhoI restriction enzymes. The resulting recombinant plasmids were named pET28-1700, pET28-1400, pET28-500, and pET28-64. The 1400 nt target sequence was generated by splicing the cleaved IRES with the GFP sequence (Figure 1B). The 64 nt sequence was composed of a 25 bp *jnk* sequence, a 9 bp loop sequence, and a 30 bp anti-*jnk* sequence (Figure 1B). As shown in Figure 1B, a circRNA and a linear sequence were synthesized if the group II intron successfully performed the self-splicing function.

To validate the correct synthesis of circRNAs using the four constructed plasmids mentioned above, the recombinant plasmids were first linearized using the Xho I enzyme. Subsequently, linear RNA molecules were obtained through in vitro transcription. The linear transcription products were then incubated at 53 °C in a circularization buffer (100 mM MgCl, 0.5 M NH_4_Cl) to allow the group II intron to self-fold into the correct secondary structure. Under the action of ribonucleases, the target segments were circularized to form circRNAs (cRNA-1700, cRNA-1400, cRNA-500, cRNA-Dumbbell). The results shown in Figure 1C indicate that all four different-sized RNA segments were successfully circularized, which resulted in distinct bands of circRNA compared with the pre-circularized state.

**Figure 2:**
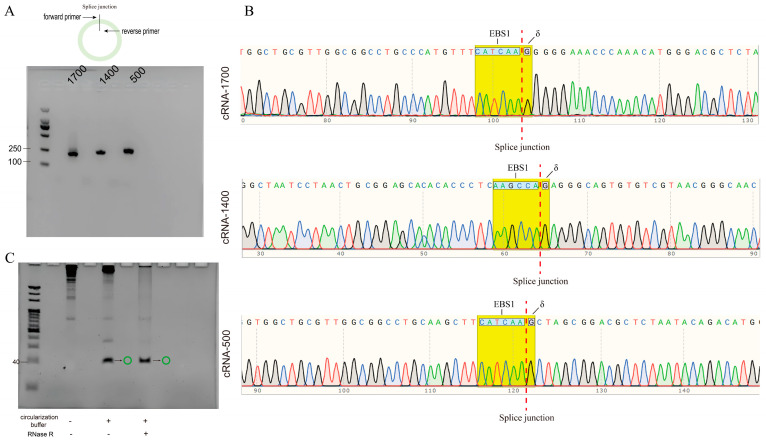
The circularization of four circRNAs was successful. (**A**) cRNA−1700, cRNA−1400, and cRNA−500 were verified by RT−PCR. 1700, ‘group of cRNA−1700’; 1400, ‘group of cRNA−1400’; 500, ‘group of cRNA−500’. (**B**) The splice junction of cRNA−1700, cRNA−1400, and cRNA−500 was connected. EBS1, ‘EBS1 sequence which complements the EBS1 of group II intron’; δ, ‘δ base which complements the δ base of group II intron’. (**C**) cRNA−Dumbbell was protected from RNase R digestion. ‘−’, ‘no added’; ‘+’, ‘added’; green ring symbol, ‘circRNA’. Original images can be found in Supplementary Materials.

To demonstrate the precision of group II intron splicing, the validation of the circularization of the 1700, 1400, and 500 nt circRNAs was performed using RT−PCR. The sequencing results confirmed the correct connection of the 3′ and 5′ ends of these three RNAs without introducing any mutations (Figure 2A,B). If cyclization was not successful, only the band of the primer dimer was shown. Due to the small size of the 64 nt RNA segment, sequencing validation after circularization was challenging when using RT−PCR. However, the 64 nt segment was resistant to RNase R digestion post-circularization, which proved that this segment could also undergo circularization using the group II intron to form circRNA (Figure 2C).

### 3.2. Synthesizing Four circRNAs Using E. coli

**Figure 3:**
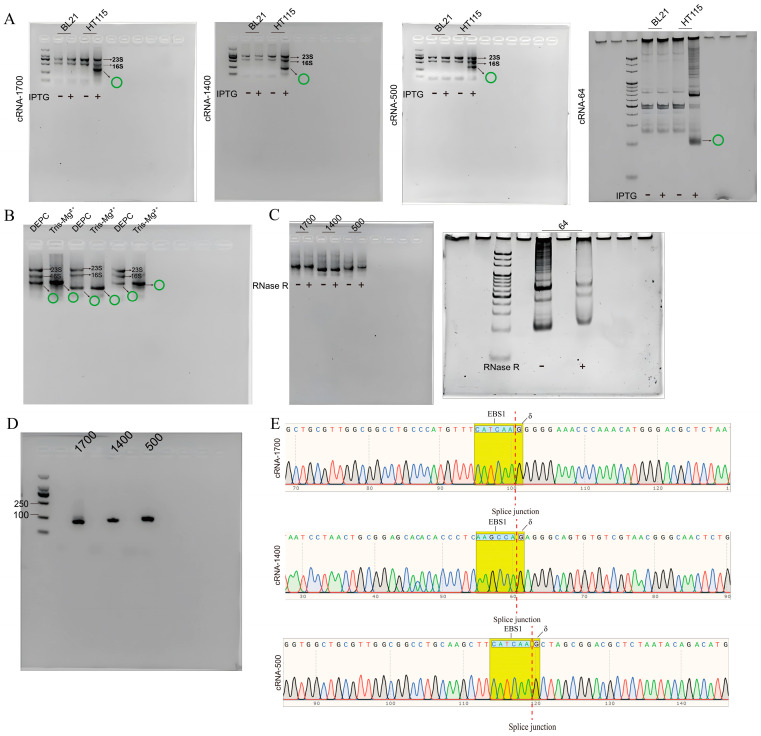
Construction and validation of circRNA production system in vivo. (**A**) The pET28−1700, pET28−1400, pET28−500, pET28−64 expression vector was expressed in HT115 (DE3). BL21, ‘BL21 (DE3)’; HT115, ‘HT115 (DE3)’; IPTG, ‘Isopropyl β−D−Thiogalactoside’; ‘−’, ‘no added’; ‘+’, ‘added’; green ring symbol, ‘circRNA’; 23 S, ‘23 S rRNA of *E. coli*’; 16 S, ‘16 S rRNA of *E. coli*’. (**B**) CircRNA was effectively enriched withTris−Mg^2+^ as the RNA extract. DEPC, ‘DEPC as the RNA extract’; Tris−Mg^2+^, ‘Tris−Mg^2+^ as the RNA extract’. (**C**) CircRNA produced in vivo was protected from RNase R digestion. cRNA−1700, cRNA−1400, cRNA−500 and cRNA−Dumbbell RNaseR digestion; ‘−’, ‘no added’; ‘+’, ‘added’. (**D**) cRNA−1700, cRNA−1400, and cRNA−500 were verified by RT−PCR. 1700, ‘group of cRNA−1700’; 1400, ‘group of cRNA−1400’; 500, ‘group of cRNA−500’. (**E**) The splice junction of cRNA−1700, cRNA−1400, and cRNA−500 was connected. EBS1, ‘EBS1 sequence which complements the EBS1 of group II intron’; δ, ‘δ base which complements the δ base of group II intron’. Original images can be found in Supplementary Materials.

To investigate the feasibility of synthesizing the aforementioned circRNAs using *E.coli*, four recombinant plasmids were transformed into two strains of *E.coli*: BL21 (DE3) [26] and HT115 (DE3) [27]. Following the cultivation, the expressions of the circRNAs were induced using IPTG for 4 h. The results depicted in Figure 3A reveal that BL21 (DE3) with the T7 RNase polymerase strain exhibited only ribosomal RNA, specifically 23s rRNA, 16s rRNA, and 5s rRNA, with no additional bands detected. In contrast, in the HT115 (DE3) strain, which has an RNase III deficiency and λ phage DE3 region, overexpressed RNA accumulated within the strain. As expected, the group of HT115 (DE3) displayed an extra RNA band, in addition to the three ribosomal RNAs. A grayscale analysis of the circRNAs using ImageJ software indicated that the circRNAs accounted for approximately 50% of the total RNA.

To improve the efficiency of the circular RNA preparation, RNA extraction was performed using a buffer with a pH of 8.0 to degrade most of the linear rRNA (Figure 3B). Additionally, further purification of the circRNA was achieved by using LiCl. The circularity and sequence integrity of the target RNA were validated through RNase R digestion (Figure 3C), reverse-transcription PCR (RT−PCR, Figure 3D), and DNA sequencing (Figure 3E). These results confirmed that the circRNA was successfully synthesized in *E. coli* HT115 (DE3) without any mutations or the introduction of additional sequences. Moreover, the use of a Tris solution with a pH of 8.0, in combination with LiCl precipitation, resulted in a higher circRNA purity.

### 3.3. Optimization of circRNA Synthesis Efficiency In Vivo

**Figure 4:**
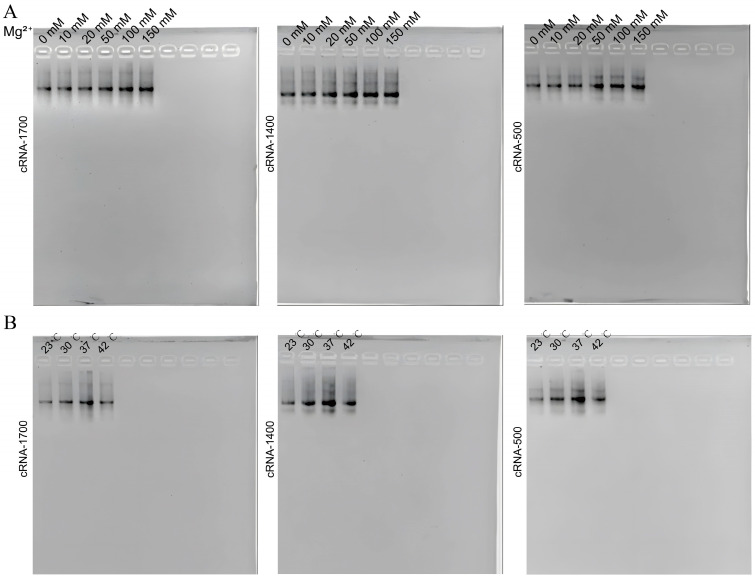
Optimization of circRNA production. (**A**) CircRNA was produced efficiently in LB, including 100 mM Mg^2+^. cRNA−1700, cRNA−1400 and cRNA−500 expression in LB with different Mg^2+^ concentrations (0 mM, 10 mM, 20 mM, 50 mM, 100 mM, and 150 mM). (**B**) CircRNA was produced efficiently at a 37 °C induction temperature. cRNA−1700, cRNA−1400 and cRNA−500 expression at different induction temperatures (23 °C, 30 °C, 37 °C, and 42 °C). Original images can be found in Supplementary Materials.

To further enhance the synthesis efficiency of the circular RNA in *E. coli*, the effects of different cultivation temperatures (23, 30, 37, and 42 °C) and Mg^2+^ concentrations in the LB medium (10, 20, 50, 100, 150 mM) on the circRNA synthesis were investigated. All the bacteria were expanded to an OD600 of 0.6 (i.e., late exponential growth phase) to ensure the same number of bacteria. The same volume of the extract was analyzed using an agarose gel. The RNA extraction, purification, and ImageJ grayscale analysis results demonstrate that the circRNA yield increased with higher Mg^2+^ concentrations, where they reached a maximum with 100 mM Mg^2+^ (Figure 4A). Additionally, the yield induced at 37 °C in the LB medium with 100 mM Mg^2+^ (Figure 4B) reached 6.89 ± 1.62 μg/10 mL of the bacterial liquid. Subsequently, ImageJ was used to analyze the grayscale of the optimized circRNA, with a maximum circularization rate of 83 ± 7%.

### 3.4. CircRNA Expressed Protein and Showed Low Cytotoxicity in Cells

**Figure 5:**
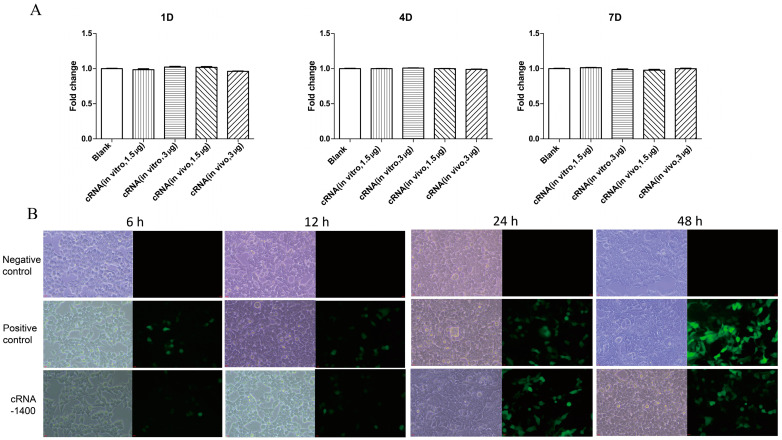
Cytotoxicity and protein expression of circRNAs. (**A**) Cell viability of Vero cells transfected with cRNA−Dumbbell compounded in vitro and in vivo. The cell viability was measured 1, 4, and 7 days after transfection, respectively. (**B**) The similar fluorescence expression abilities of cRNA−1400 produced in vitro and cRNA−1400 produced in vivo were demonstrated at 6–48 h. Negative control, ‘transfected DEPC water group’; positive control, ‘transfected cRNA−1400 produced in vitro group’; cRNA−1400, ‘transfected cRNA−1400 produced in vitro group’; green fluorescence, 'GFP protein'.

To confirm that the one-step synthesized circRNA was functional for RNA vaccine and RNA silencing applications, the circRNAs derived from the pET28−1400 and pET28−64 were transfected into 293 T and Vero cells, respectively. The 64 nt circRNA transcribed in vitro served as the positive control, while DEPC water served as the negative control. The results show that on the seventh day of the RNA processing, compared with the control group, no toxicity was observed (Figure 5A). The 1400 nt circRNA transcribed in vitro was the positive control and DEPC water was used as the negative control. The expression of GFP was observed under a fluorescence microscope at 6, 12, 24, and 48 h post-transfection. The results show that, similar to the circRNA prepared using in vitro transcription, the GFP protein was also expressed in the 293 T cells, which were transfected with circRNA synthesized using an *E. coli* one-step method within 48 h. This indicates that this method can be used in the field of RNA vaccines, which will significantly reduce the preparation cost and technical difficulties of circRNA (Figure 5B).

### 3.5. CRNA−Dumbbell Reduced Target Gene Expression and Induced Apoptosis in Hep 3B Cells

**Figure 6:**
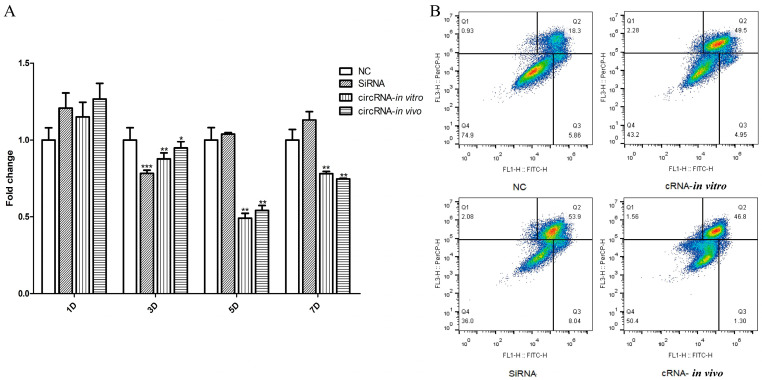
cRNA−Dumbbell induced the downregulation of target genes and apoptosis. (**A**) The downregulation rate of *jnk* expression in Hep 3B cells was similar in both circRNA−in vitro and circRNA−in vivo groups. (**B**) The apoptosis rate of Hep 3B cells in the circRNA−in vitro and circRNA−in vivo groups was similar. NC, ‘transfected DEPC water group’; SiRNA, ‘transfected siRNA group’; cRNA−in vitro; ‘transfected cRNA− Dumbbell produced group in vitro; cRNA−in vivo; ‘transfected cRNA− Dumbbell group produced in vivo. ‘*’, ‘*p* < 0.05. ‘**’, ‘*p* < 0.01’, ‘***’, ‘*p* < 0.001.

The RNAi effect of cRNA−Dumbbell was assessed using qPCR and flow cytometry. The DEPC water was used as the negative control and siRNA was the positive control. The results show that the RNAi effect of the cRNA−Dumbbell transcribed in vitro and produced in vivo were almost the same, and five days after the transfection, the target gene expression was reduced by the circRNA (Figure 6A). Apoptosis was detected three days after the transfection; the apoptosis rates of the DEPC water, siRNA, and cRNA−Dumbbell transcribed in vitro and produced in the in vivo groups were 24.16, 61.94, 54.45, and 48.1%, respectively (Figure 6B).

## 4. Discussion

Both domestically and internationally, most researchers currently use cells or bacteria to achieve the in vivo cyclization of RNA. HEK293T cells are commonly used as the main cell line for in vivo circRNA production. HEK293T cells exhibited a high yield compared with HeLa, B16, and HepG2 cells, where they reached up to 21 μM [10,17]; this may have been because of the expression of the temperature-sensitive allele of the SV40T antigen, which amplifies the vector containing the SV40 ori, thereby significantly increasing its transient transfection expression levels. Additionally, studies showed that even after multiple freeze–thaw cycles and passages, HEK293T cells still exhibited high stability, which provides a solid foundation for large-scale circRNA production [28]. In addition, bacteria were also used for large-scale in vivo circRNA production due to their lower cost, operational simplicity, and sustainable production advantages. The most-used bacteria for in vivo production are BL21 (DE3) strains [26,29]. However, in BL21 cells, the precursor circular RNA is rapidly degraded by RNase III after transcription, which leads to the disappearance of circular RNA within 24 hours, thus making it difficult for it to accumulate in large quantities within the cells. Additionally, studies showed that BL21 cells are unable to produce short circRNA fragments with hairpin structures (40–100 nt), while this was achieved in HT115 cells [30]. Therefore, in this experiment, HT115 (DE3) cells were chosen as the host for the in vivo circRNA production. HT115 (DE3) cells have a defect in RNase III that prevents the rapid degradation of exogenous RNA after its induced expression, thus allowing for self-splicing under the action of group II introns to form stable circular RNA. Utilizing the splicing mechanisms of different ribonucleases for the in vivo production of circRNA, as opposed to in vitro synthesis, offers many advantages, such as a low cost, operational ease, and a low immunogenicity; accordingly, it has attracted widespread attention, both domestically and internationally. However, this research field started relatively late, and many ribonuclease circularization mechanisms are still not clear, which poses significant challenges in the field of production application. The group I intron was used to circularize RNA in vitro or in vivo, whether in cells or *E. coli* (BL21) [20,26,29,31,32]. However, most of the research on in vivo circularization using this intron focused on sequences between 70 and 1000 nt in length; exceeding this range significantly reduced the circularization rate. Moreover, the group I intron elements that were used for the in vivo circularization still introduced exogenous sequences, which could trigger immune reactions in the organism, thereby limiting the application of in vivo-produced circRNA in mRNA vaccines or RNAi fields. Therefore, the future exploration of in vivo circularization should tend toward broader circularization ranges and scarless precise splicing. In recent years, as ribonuclease mechanism studies continued in more depth, a greater variety of ribonucleases are being used for in vivo sequence circularization. Actin introns can be used as circularization elements with a much higher circularization rate than the T4 bacteriophage *Td* gene’s group I intron splicing system, but the intron sequence may bind to inhibitory factors, which limits its splicing ability [10]. Furthermore, ALU elements circularized sequences as short as 51 nt intracellularly without introducing any exogenous sequences, but this method requires compatibility between the circularization sequence and the intron sequence and leads to unwanted byproducts due to the competitive splicing reactions in vivo, which increases the purification costs [15]. Additionally, hammerhead ribozymes [30] and hairpin ribozymes [33] mediated the circularization of short sequences that ranged from 40 to 100 nt in *E. coli* without introducing any exogenous sequences, which is suitable for the in vivo mass production of sciRNAs, but these ribonucleases cannot be designed for circularizing long sequences and cannot sustain continuous production. This study used the *Clostridium tetani* group II intron, which was verified to precisely circularize sequences that ranged from 1300 to 10,000 nt in vitro by reverse-splicing the intron sequence and utilizing the sequence recognition at the EBS1−IBS1 and δ−δ’ sequence ends without introducing any exogenous sequences. The in vitro synthesis of anti-tumor circRNA vaccines utilizing group II introns has demonstrated substantial efficacy via the intracellular expression of proteins. The cellular immune response was enhanced due to the potent adjuvant properties of the circRNA vaccine. As such, a broad application prospect is expected in the production and development of circRNA vaccines. In this study, the in vitro and in vivo syntheses of fluorescently expressing cRNA−1400 were demonstrated to be effective in expressing fluorescent proteins. The present study offers a promising novel approach for the development of circRNA vaccines. In addition, no reports currently exist of this intron element being used in short-sequence circularization. Naoko Abe found that different stem–loop ratios affected the efficiency of RNAi in the exploration of dumbbell-shaped sciRNA and, by evaluating its silencing efficiency and stability in vivo, concluded that the most effective combination for siRNA design was a 23 bp stem with 9 nt loops at both ends [6]. Therefore, based on the *Clostridium tetani* group II intron element, four circular RNA vectors (pET28−1700, pET28−1400, pET28−500, and pET28−64) were designed, for mRNA and siRNA applications; this enabled the precise circularization of any sequence within the 64–1700 nt range in vivo and in vitro without introducing any exogenous sequences and requiring no additional circularization steps besides purification using LiCl precipitation, with a yield of up to 820 ng/mL of bacterial liquid. It is worth mentioning that a study on sciRNAs found that sciRNAs with a 27 nt circular sense chain and a 21 nt linear antisense chain exhibited the highest stability and silencing efficiency [34]. As the intron element used in this study did not introduce exogenous sequences and was designed for the precise circularization of any sequence as needed, this study also designed a circular sciRNA with a 27 bp circular sense chain named pET28−27 and investigated its circularization in vivo and in vitro. The results show that it was circularized in vitro (Figure S2), but was not detected in vivo, which was probably because of the extremely low yield in *E. coli*.

Using ribozymes, circRNAs of different lengths were cyclized in vivo. However, the yield of the cyclized circRNA was also affected by the length of the cyclization sequence. The method, based on the group I intron, demonstrated that the yield of the circRNA decreased with increasing length when the sequence length of the circRNA was larger than 1200 nt [12], and when the fragment length was shorter than 300 nt, the yield of the circRNA decreased with decreasing length [30]. This study explored the cyclization sequence length of the group II intron elements and found that the cyclization efficiency decreased as the sequence length decreased when the cyclization sequence length was less than 1700 nt. This result was consistently observed between the in vitro and in vivo cases. It is worth mentioning that the production methods currently used for the in vivo production of sciRNA mostly suffer from a low yield and the inability to sustain production when using short cyclization sequences. For example, using hammerhead ribozymes to produce circRNA of 83–103 nt in length only achieved a cyclization efficiency of 6.7–9.5% [33]. In contrast, this study’s cyclization system achieved a cyclization efficiency of approximately 50% for the 64 nt circRNA after optimization, which provides a feasible solution for the in vivo cyclization of short sequences.

The splicing mechanism of group II introns was difficult to study because of their complex structure and extensive classification, which limits their application in various fields. Currently, research on the application of group II introns is rare, with the main focus being on their use in gene targeting through the retrohoming mechanism [35]. In this study, we used the group II intron C.te.I1 from the *Clostridium tetani* surface protein and constructed a system for the large-scale production of circRNA in *E. coli* HT115 (DE3) cells. We systematically investigated the optimal cyclization conditions and extraction methods. For the in vitro cyclization conditions of this intron (which only required Mg^2+^), we explored the optimal ion environment by setting different final concentrations of the Mg^2+^ in the LB medium, and the same expected Mg^2+^ dependence was found. Additionally, group II introns were defined as thermophilic introns. In this study, we referred to the induction temperature (42 °C) used in the reported applications of group II introns for in vivo gene targeting [36], the normal induction temperature of *E. coli* (37 °C), the induction temperature for the in vivo cyclization of group I introns (30 °C) [37], and the lowest exploratory temperature (23 °C) to systematically investigate the highest-yielding temperature. The highest yield of circRNA was produced at 37 °C, which may have been because the essential factor was the RNA polymerase of *E. coli*, which has a maximum efficiency at 37 °C for this method, rather than because of the function of the type II intron itself (homing effect).

## 5. Conclusions

In summary, in this study, the circular RNA synthesis process was simplified, and the circRNA production costs were reduced. The steps used to produce circRNA in vitro were plasmid extraction, enzyme digestion, enzyme digestion product gel extraction, and in vitro transcript product cyclization. In this study, we synthesized circRNA with a yield of 6.26 ± 1.31 μg/300 ng plasmid using a single in vitro transcriptional cyclization. To produce circRNA in this way, at least four kits needed to be used, which increased the production cost. However, in this system, the production of circRNAs was achieved by only culturing *E. coli* to induce the expression of circRNAs, and the circRNA was extracted using the phenol–chloroform method. Remarkably, our circRNA production system produced 6.89 ± 1.62 μg/10 mL bacterial solution. Moreover, in this system, the production of circRNA was sustainable due to the preservability of *E. coli*. Furthermore, in the in vitro production method, the purified plasmid template was subjected to only one in vitro transcription reaction. The circRNA production system established in this study provided new evidence for future large-scale circRNA production and the exploration of the in vivo and in vitro splicing mechanisms of group II introns, which are worthy of further research.

## Data Availability

The original contributions presented in this study are included in the article/supplementary material. Further inquiries can be directed to the corresponding author(s).

## Supplementary Materials

The following supporting information can be downloaded at: https://www.mdpi.com/article/10.3390/biom14111416/s1, Figure S1: Flow chart of circRNA preparation; Figure S2: The digestion results of cRNA-27 with RNaseR; Table S1: Overlapping PCR primer of SC sequence; Table S2: PCR primer sequence; Table S3: RT primer sequence.
