# Supplementary material for: A Simple and Efficient One-Step Synthesis System for Flexible Production of Circular RNA in E. coli
Source: Biomolecules. 2024 Nov 7;14(11):1416. doi: 10.3390/biom14111416 (PMC11592204; doi:10.3390/biom14111416)
Supplement: Supplementary file 1 [file biomolecules-14-01416-s001.zip › biomolecules-3210784-SM.pdf]

＞1700
TAATACGACTCACTATAGGCTATTATCGAGCGAACGCCTTATGCGATGAAAGTCGCACGTAGGGTGTAGACCA
AGCGAAATCCTATGCATTTAGGATAGTGAGGTATGGGGGAAACCCAAACATGGGACGCTCTAATACAGACAT
GGTGCGAAGAGTCTATTGAGCTAGTTGGTAGTCCTCCGGCCCCTGAATGCGGCTAATCCTAACTGCGGAGCA
CACACCCTCAAGCCAGAGGGCAGTGTGTCGTAACGGGCAACTCTGCAGCGGAACCGACTACTTTGGGTGT
CCGTGTTTCATTTTATTCCTATACTGGCTGCTTATGGTGACAATTGAGAGATCGTTACCATATAGCTATTGGATT
GGCCATCCGGTGACTAATAGAGCTATTATATATCCCTTTGTTGGGTTTATACCACTTAGCTTGAAAGAGGTTAA
AACATTACAATTCATTGTTAAGTTGAATACAGCAAAACTAGTGCCACcATGGATGCAATGAAGAGAGGGCTC
TGCTGTGTGCTGCTGCTGTGTGGAGCAGTCTTCGTTTCGCCCAGCCAGGAAATCCATGCCCGATTCAGAAGA
CGCGTGAGCAAGGGCGAGGAGCTGTTCACCGGGGTGGTGCCCATCCTGGTCGAGCTGGACGGCGACGTA
AACGGCCACAAGTTCAGCGTGTCCGGCGAGGGCGAGGGCGATGCCACCTACGGCAAGCTGACCCTGAaGT
TCATCTGCACCACCGGCAAGCTGCCCGTGCCCTGGCCCACCCTCGTGACCACCCTGACCTACGGCGTGCAGT
GCTTCAGCCGCTACCCCGACCACATGAAGCAGCACGACTTCTTCAAGTCCGCCATGCCCGAAGGCTACGTCC
AGGAGCGCACCATCTTCTTCAAGGACGACGGCAACTACAAGACCCGCGCCGAGGTGAAGTTCGAGGGCGA
CACCCTGGTGAACCGCATCGAGCTGAAGGGCATCGACTTCAAGGAGGACGGCAACATCCTGGGGCACAAG
CTGGAGTACAACTACAACAGCCACAACGTCTATATCATGGCCGACAAGCAGAAGAACGGCATCAAGGTGAA
CTTCAAGATCCGCCACAACATCGAGGACGGCAGCGTGCAGCTCGCCGACCACTACCAGCAGAACACCCCCA
TCGGCGACGGCCCCGTGCTGCTGCCCGACAACCACTACCTGAGCACCCAGTCCGCCCTGAGCAAAGACCCC
AACGAGAAGCGCGATCACATGGTCCTGCTGGAGTTCGTGACCGCCGCCGGGATCACTCTCGGCATGGACGA
GCTGTACAAGGGTACCCAATCGCAAGCTTGGCGGAGGAGGCAGCGGCGGAGGAGGCAGCGGAAGCGGC
TACATCCCAGAAGCCCCTAGAGACGGACAGGCTTACGTGCGAAAAGACGGCGAGTGGGTGCTGCTGAGCA
CATTCCTGGGAAGGAGCTGATGATGAGGGTTTTTAAAACAGCCTGTGGGTTGATCCCACCCACAGGCCCAT
TGGGCGCTAGCACTCTGGTATCACGGTACCTTTGTGCGCCTGTTTTATACCCCCTCCCCCAACTGTAACTTAG
AAGTAACACACACCGATCAACAGTCAGCGTGGCACACCAGCCACGTTTTGATCAAGCACTTCTGTTACCCCG
GACTGAGTATCAATAGACTGCTCACGCGGTTGAAGGAGAAAGCGTTCGTTATCCGGCCAACTACTTCGAAA
AACCTAGTAACACCGTGGAAGTTGCAGAGTGTTTCGCTCAGCACTACCCCAGTGTAGATCAGGTCGATGAGT
CACCGCATTCCCCACGGGCGACCGTGGCGGTGGCTGCGTTGGCGGCCTGCCCATGTTTCATCAACAATAAA
AGTGCGAAACGTTATCCTATAAGTAAGAAAGTTTTAAAATTTTCTTACGAAAAGGATAGAACTTAAAAGTTCT
AACTGTTCTACTAAAGTAATAAGTGAAAATCTTATTTAAAGCAAACAACCAAGTAGCTTTAAGTCTAAGTCCC
CTACACAAGTTTTATACTACTATGCAAAACTTGTGAAGCTAGGTAAGGTCGTAATCCGTGAAAGTCGGATGCG
GGGCTCCTTAAAAGATTACTATGGTAAACATAAGCTAATCCATTAAGATGCGATTTATATGTATTTTATACTGTT
AAATATTTTTGTGCCTTGATGTTGGTATAAAACAGTTAAGATGAAGTACTTAACTGGTTTTGGAATAATTGGTT
GTTAAACTAAAACATTATAAATCGTTAGTGGATACCTAAGGTAATCAAAAATAGGGATAGGTAGAATGGAACG
TTTGATGCTGTATATGAAGAGGTTTAGTAGAACCTAGGACACATATACGGGCTCAGCAGGTTCATAGTAGCTA
TGATACTCAGCCGGAAGTCAAATTAATTTTGAAATACTTCTATGGTAACATAGGAGAAGGATAAAACTGAGTG
AGCCAAGGAACCTAGTCGGTAATAG

＞1400
TAATACGACTCACTATAGGCTATTATCGAGCGAACGCCTTATGCGATGAAAGTCGCACGTAGGGTGTAGACCA
AGCGAAATCCTATGCATTTAGGATAGTGAGGTATGAGGGCAGTGTGTCGTAACGGGCAACTCTGCAGCGGA
ACCGACTACTTTGGGTGTCCGTGTTTCATTTTATTCCTATACTGGCTGCTTATGGTGACAATTGAGAGATCGTT
ACCATATAGCTATTGGATTGGCCATCCGGTGACTAATAGAGCTATTATATATCCCTTTGTTGGGTTTATACCACT
TAGCTTGAAAGAGGTTAAAACATTACAATTCATTGTTAAGTTGAATACAGCAAAGCCACCATGGTGAGCAAG
GGCGAGGAGCTGTTCACCGGGGTGGTGCCCATCCTGGTCGAGCTGGACGGCGACGTAAACGGCCACAAG

TTCAGCGTGTCCGGCGAGGGCGAGGGCGATGCCACCTACGGCAAGCTGACCCTGAAGTTCATCTGCACCAC
CGGCAAGCTGCCCGTGCCCTGGCCCACCCTCGTGACCACCCTGACCTACGGCGTGCAGTGCTTCAGCCGCT
ACCCCGACCACATGAAGCAGCACGACTTCTTCAAGTCCGCCATGCCCGAAGGCTACGTCCAGGAGCGCACC
ATCTTCTTCAAGGACGACGGCAACTACAAGACCCGCGCCGAGGTGAAGTTCGAGGGCGACACCCTGGTGA
ACCGCATCGAGCTGAAGGGCATCGACTTCAAGGAGGACGGCAACATCCTGGGGCACAAGCTGGAGTACAA
CTACAACAGCCACAACGTCTATATCATGGCCGACAAGCAGAAGAACGGCATCAAGGTGAACTTCAAGATCC
GCCACAACATCGAGGACGGCAGCGTGCAGCTCGCCGACCACTACCAGCAGAACACCCCCATCGGCGACGG
CCCCGTGCTGCTGCCCGACAACCACTACCTGAGCACCCAGTCCGCCCTGAGCAAAGACCCCAACGAGAAGC
GCGATCACATGGTCCTGCTGGAGTTCGTGACCGCCGCCGGGATCACTCTCGGCATGGACGAGCTGTACAAG
TGATGATTAAAACAGCCTGTGGGTTGATCCCACCCACAGGCCCATTGGGCGCTAGCACTCTGGTATCACGGT
ACCTTTGTGCGCCTGTTTTATACCCCCTCCCCCAACTGTAACTTAGAAGTAACACACACCGATCAACAGTCAG
CGTGGCACACCAGCCACGTTTTGATCAAGCACTTCTGTTACCCCGGACTGAGTATCAATAGACTGCTCACGC
GGTTGAAGGAGAAAGCGTTCGTTATCCGGCCAACTACTTCGAAAAACCTAGTAACACCGTGGAAGTTGCAG
AGTGTTTCGCTCAGCACTACCCCAGTGTAGATCAGGTCGATGAGTCACCGCATTCCCCACGGGCGACCGTGG
CGGTGGCTGCGTTGGCGGCCTGCCCATGGGGAAACCCATGGGACGCTCTAATACAGACATGGTGCGAAGA
GTCTATTGAGCTAGTTGGTAGTCCTCCGGCCCCTGAATGCGGCTAATCCTAACTGCGGAGCACACACCCTCA
AGCCACAATAAAAGTGCGAAACGTTATCCTATAAGTAAGAAAGTTTTAAAATTTTCTTACGAAAAGGATAGA
ACTTAAAAGTTCTAACTGTTCTACTAAAGTAATAAGTGAAAATCTTATTTAAAGCAAACAACCAAGTAGCTTTA
AGTCTAAGTCCCCTACACAAGTTTTATACTACTATGCAAAACTTGTGAAGCTAGGTAAGGTCGTAATCCGTGA
AAGTCGGATGCGGGGCTCCTTAAAAGATTACTATGGTAAACATAAGCTAATCCATTAAGATGCGATTTATATGT
ATTTTATACTGTTAAATATTTTTGTGCCTGGCTTTTGGTATAAAACAGTTAAGATGAAGTACTTAACTGGTTTT
GGAATAATTGGTTGTTAAACTAAAACATTATAAATCGTTAGTGGATACCTAAGGTAATCAAAAATAGGGATAG
GTAGAATGGAACGTTTGATGCTGTATATGAAGAGGTTTAGTAGAACCTAGGACACATATACGGGCTCAGCAG
GTTCATAGTAGCTATGATACTCAGCCGGAAGTCAAATTAATTTTGAAATACTTCTATGGTAACATAGGAGAAG
GATAAAACTGAGTGAGCCAAGGAACCTAGTCGGTAATAG

＞500
TAATACGACTCACTATAGGCTATTATCGAGCGAACGCCTTATGCGATGAAAGTCGCACGTAGGGTGTAGACCA
AGCGAAATCCTATGCATTTAGGATAGTGAGGTATGCTAGCGGACGCTCTAATACAGACATGGTGCGAAGAGT
CTATTGAGCTAGTTGGTAGTCCTCCGGCCCCTGAATGCGGCTAATCCTAACTGCGGAGCACACACCCTCAAG
CCAGAGGGCAGTGTGTCGTAACGGGCAACTCTGCAGCGGAACCGACTACTTTGGGTGTCCGTGTTTCATTT
TATTCCTATACTGGCTGCTTATGGTGACAATTGAGAGATCGTTACCATATAGCTATTGGATTGGCCATCCGGTG
ACTAATAGAGCTATTATATATCCCTTTGTTGGGTTTATACCACTTAGCTTGAAAGAGGTTAAAACATTACAATCA
CTTCTGTTACCCCGGACTGAGTATCAATAGACTGCTCACCTAGTAACACCGTGGAAGTTGCAGAGTGTTTCG
CTCAGCACTACCCCAGTGTAGATCAGGTCGATGAGTCACCGCATTCCCCACGGGCGACCGTGGCGGTGGCT
GCGTTGGCGGCCTGCAAGCTTCATCAACAATAAAAGTGCGAAACGTTATCCTATAAGTAAGAAAGTTTTAAA
ATTTTCTTACGAAAAGGATAGAACTTAAAAGTTCTAACTGTTCTACTAAAGTAATAAGTGAAAATCTTATTTAA
AGCAAACAACCAAGTAGCTTTAAGTCTAAGTCCCCTACACAAGTTTTATACTACTATGCAAAACTTGTGAAGC
TAGGTAAGGTCGTAATCCGTGAAAGTCGGATGCGGGGCTCCTTAAAAGATTACTATGGTAAACATAAGCTAA
TCCATTAAGATGCGATTTATATGTATTTTATACTGTTAAATATTTTTGTGCCTTGATGTTGGTATAAAACAGTTAA
GATGAAGTACTTAACTGGTTTTGGAATAATTGGTTGTTAAACTAAAACATTATAAATCGTTAGTGGATACCTAA
GGTAATCAAAAATAGGGATAGGTAGAATGGAACGTTTGATGCTGTATATGAAGAGGTTTAGTAGAACCTAGG
ACACATATACGGGCTCAGCAGGTTCATAGTAGCTATGATACTCAGCCGGAAGTCAAATTAATTTTGAAATACT
TCTATGGTAACATAGGAGAAGGATAAAACTGAGTGAGCCAAGGAACCTAGTCGGTAATAG

＞64

TAATACGACTCACTATAGGCTATTATCGAGCGAACGCCTTATGCGATGAAAGTCGCACGTAGGGTGTAGACCA
AGCGAAATCCTATGCATTTAGGATAGTGAGGTATAGGAAAAATGTGGGGTTCTTCCAAATACAAGAGATTTG
GAAGAACCCCACATTTTTCCGCCACACAATAAAAGTGCGAAACGTTATCCTATAAGTAAGAAAGTTTTAAAAT
TTTCTTACGAAAAGGATAGAACTTAAAAGTTCTAACTGTTCTACTAAAGTAATAAGTGAAAATCTTATTTAAAG
CAAACAACCAAGTAGCTTTAAGTCTAAGTCCCCTACACAAGTTTTATACTACTATGCAAAACTTGTGAAGCTA
GGTAAGGTCGTAATCCGTGAAAGTCGGATGCGGGGCTCCTTAAAAGATTACTATGGTAAACATAAGCTAATC
CATTAAGATGCGATTTATATGTATTTTATACTGTTAAATATTTTTGTGCTTGTGGCTTGGTATAAAACAGTTAAG
ATGAAGTACTTAACTGGTTTTGGAATAATTGGTTGTTAAACTAAAACATTATAAATCGTTAGTGGATACCTAAG
GTAATCAAAAATAGGGATAGGTAGAATGGAACGTTTGATGCTGTATATGAAGAGGTTTAGTAGAACCTAGGA
CACATATACGGGCTCAGCAGGTTCATAGTAGCTATGATACTCAGCCGGAAGTCAAATTAATTTTGAAATACTTC
TATGGTAACATAGGAGAAGGATAAAACTGAGTGAGCCAAGGAACCTAGTCGGTAATAG

| primer | Sequence |
|---|---|
| F1 | CCGGCGTAGAGGATCGAGATCTTAATACGACTCACTATAGGC |
| R1 | GGTGGTGGTGGTGGTGCTCGAGCTATTACCGACTAGGTTCCTTG |

Table S1 Overlapping PCR primer of SC sequence

| PCR primer | Sequence |
|---|---|
| CRNA-1700 Forward primer | AACACCGTGGAAGTTGCAG |
| CRNA-1700 Reverse primer | GGCCGGAGGACTACCAA |
| CRNA-1400 Forward primer | GGTGCGAAGAGTCTATTGAGCT |
| CRNA-1400 Reverse primer | TTAGTCACCGGATGGCCAA |
| CRNA-500 Forward primer | AGACTGCTCACCTAGTAACACCGT |
| CRNA-500 Reverse primer | GTGTGTGCTCCGCAGTTAGG |

Table S2 PCR primer sequence

| RT primer | Sequence |
|---|---|
| CRNA-1700 | TTGATGAAACATGGGCAGG |
| CRNA-1400 | TGGCTTGAGGGTGTGT |
| CRNA-500 | TTGATGAAGCTTGCAGG |

Table S3 RT primer sequence

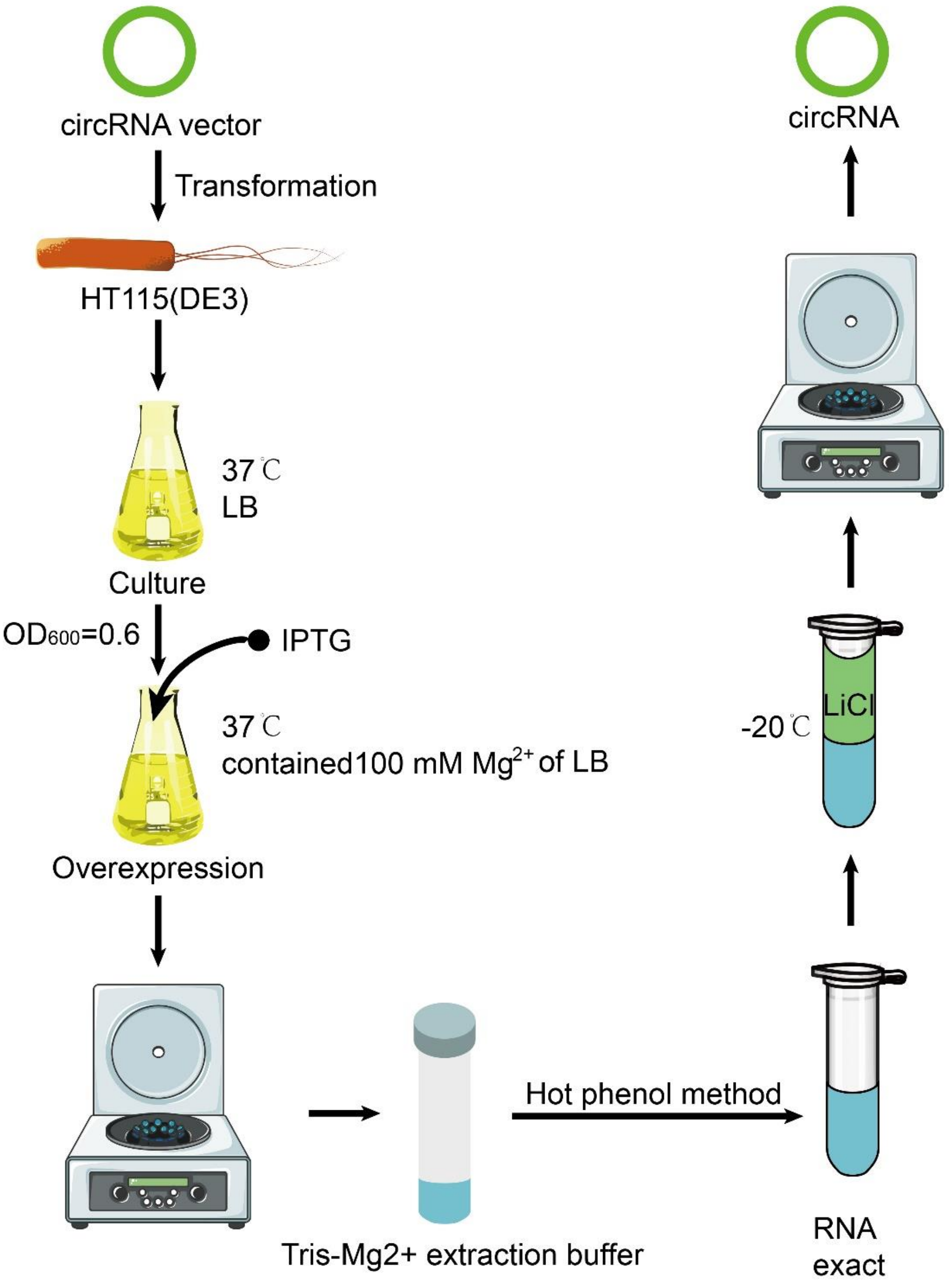


**Figure S1 Flow chart of circRNA preparation.**

M 1 2 3

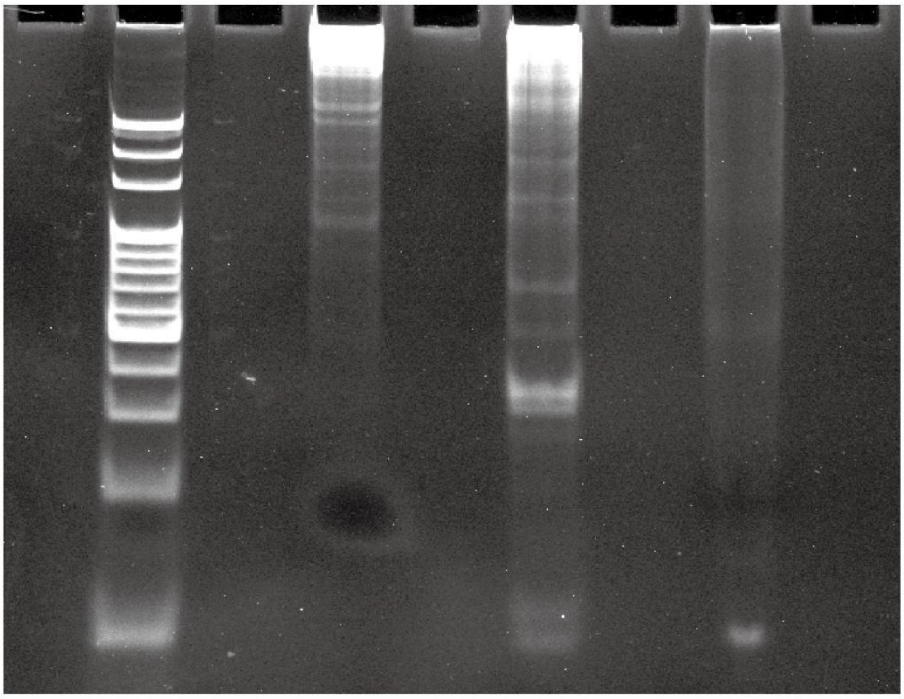

**Figure S2 The digestion results of cRNA-27 with RNaseR.** Lane M: 20bp DNA Marker, Lane 1: cRNA-27 in vitro transcription and purification, Lane 2: cRNA-27 in vitro circularization, Lane 3: cRNA-27 digestion with RNase R.
