# Supplementary material for: A Simple and Efficient One-Step Synthesis System for Flexible Production of Circular RNA in E. coli
Source: Biomolecules. 2024 Nov 7;14(11):1416. doi: 10.3390/biom14111416 (PMC11592204; doi:10.3390/biom14111416)
Supplement: Supplementary file 1 [file biomolecules-14-01416-s001.zip › original image.pdf]

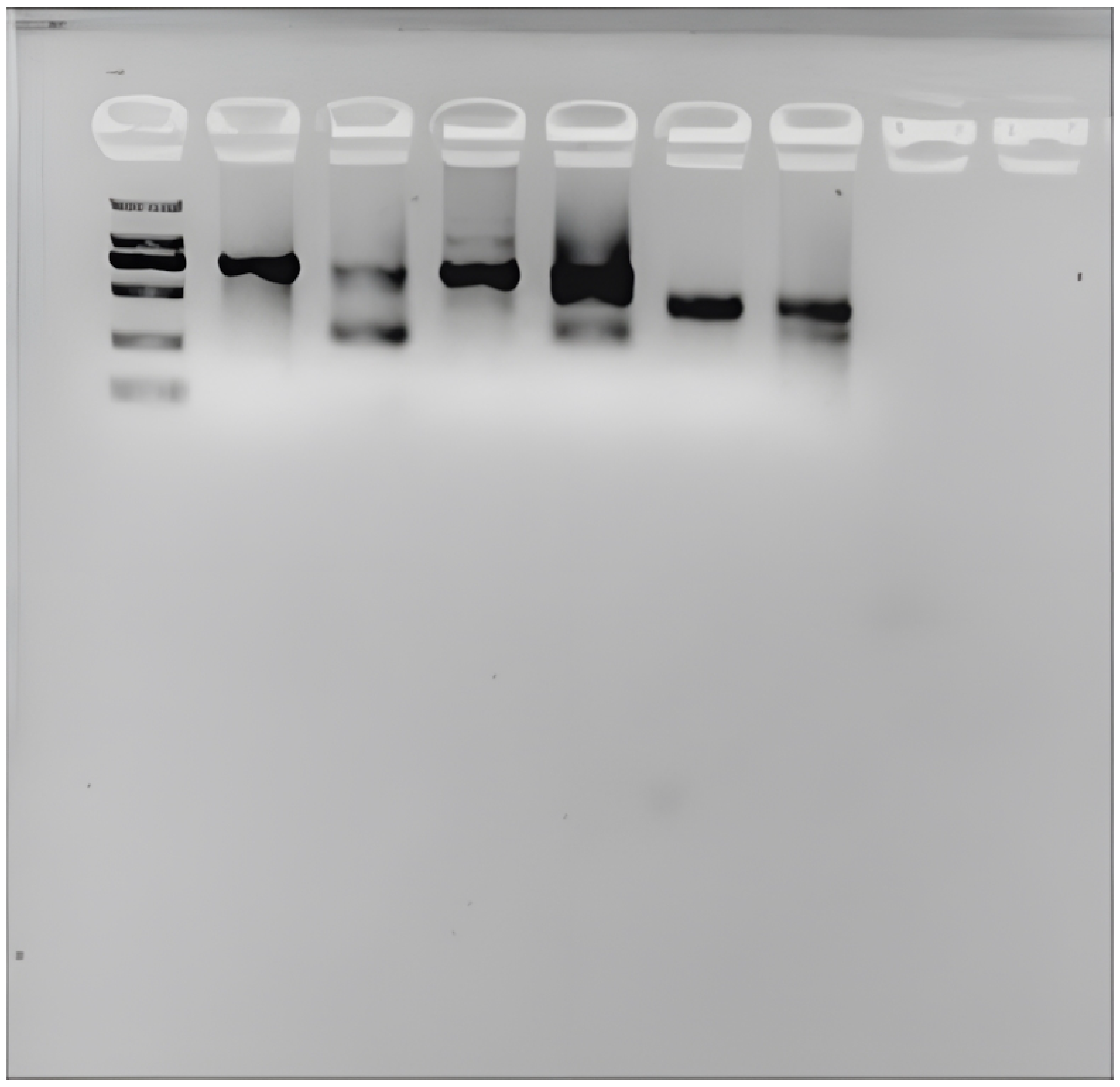

Figure 1C

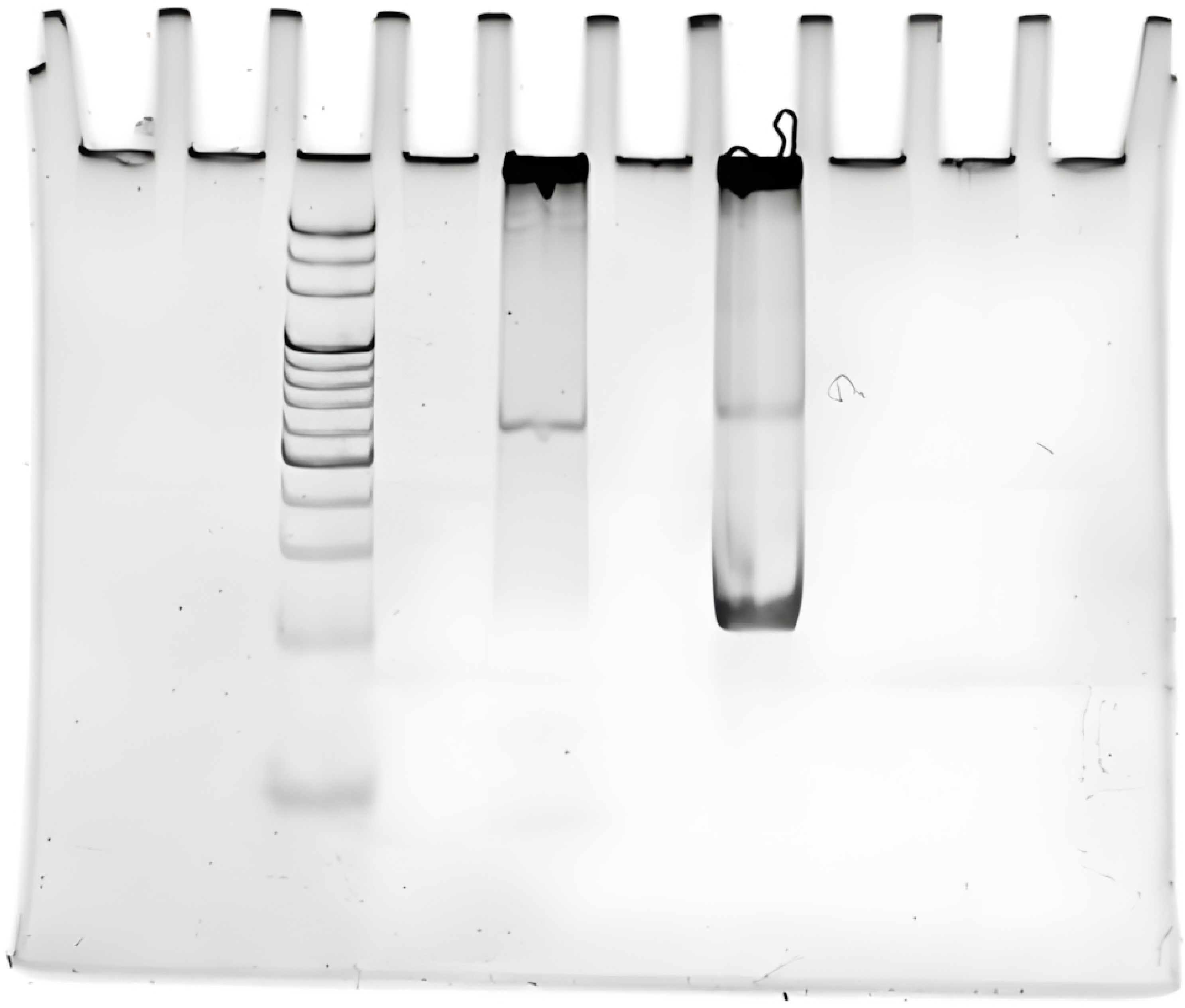

Figure 1C

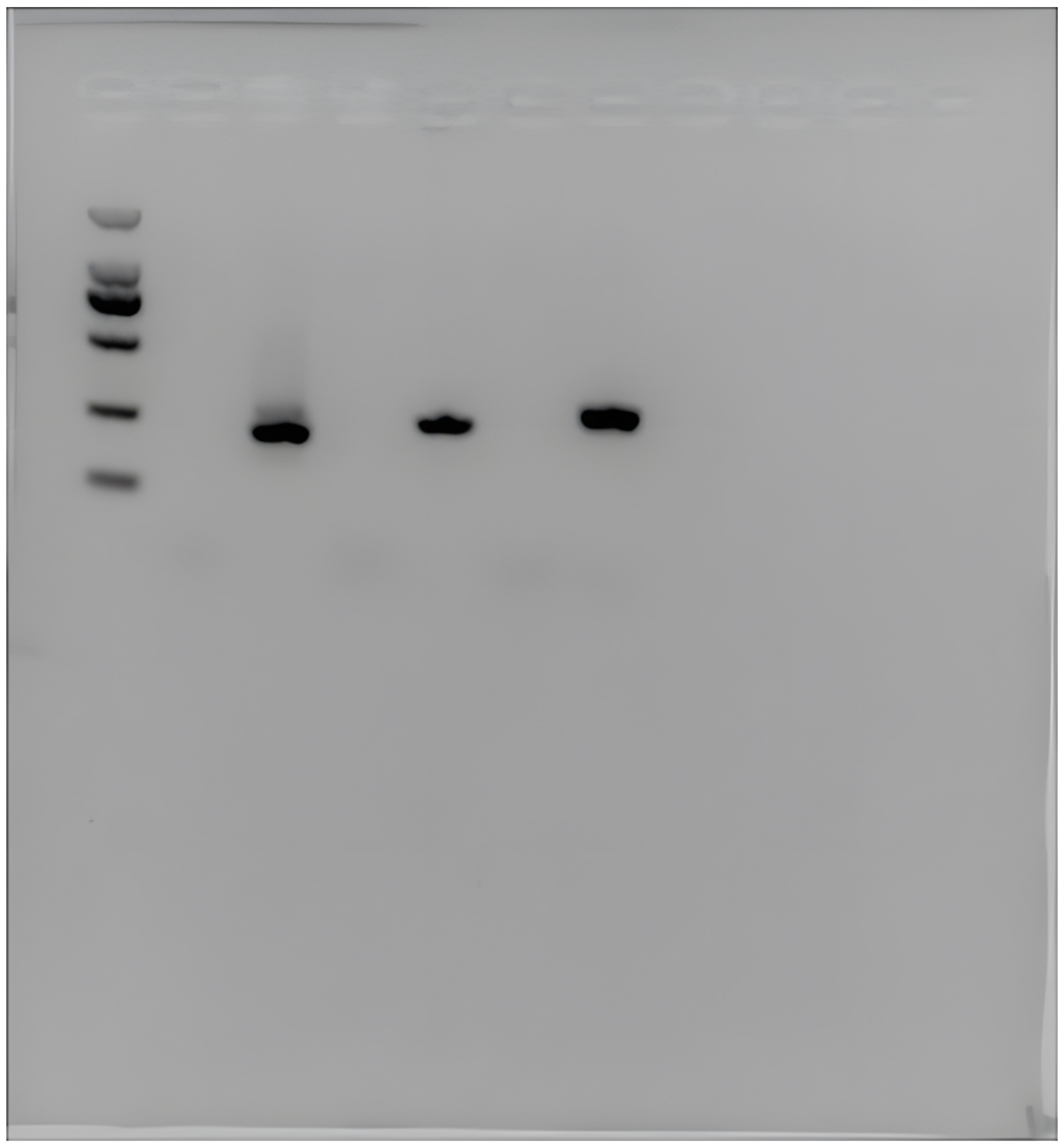

Figure 2A

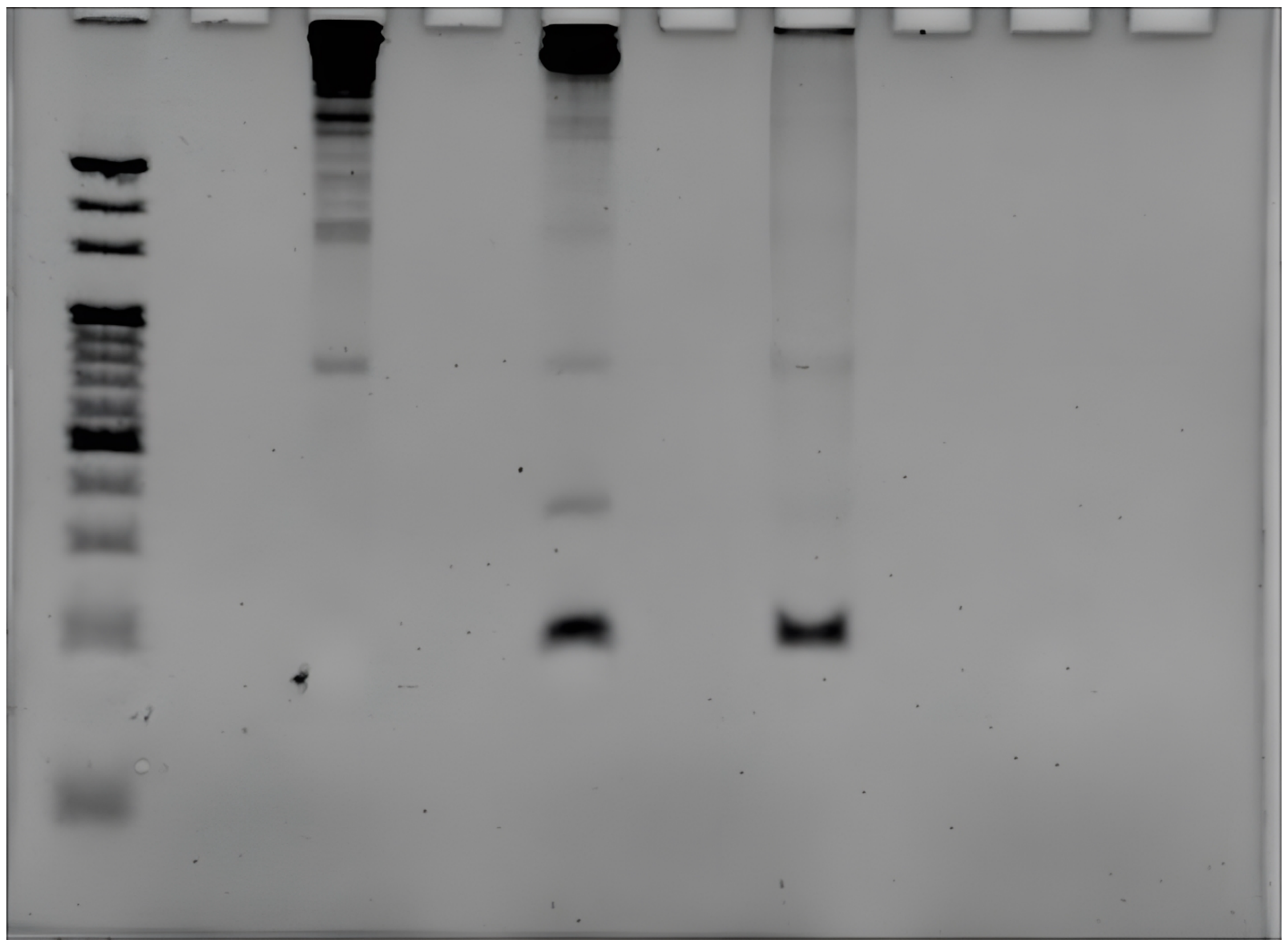

Figure 2C

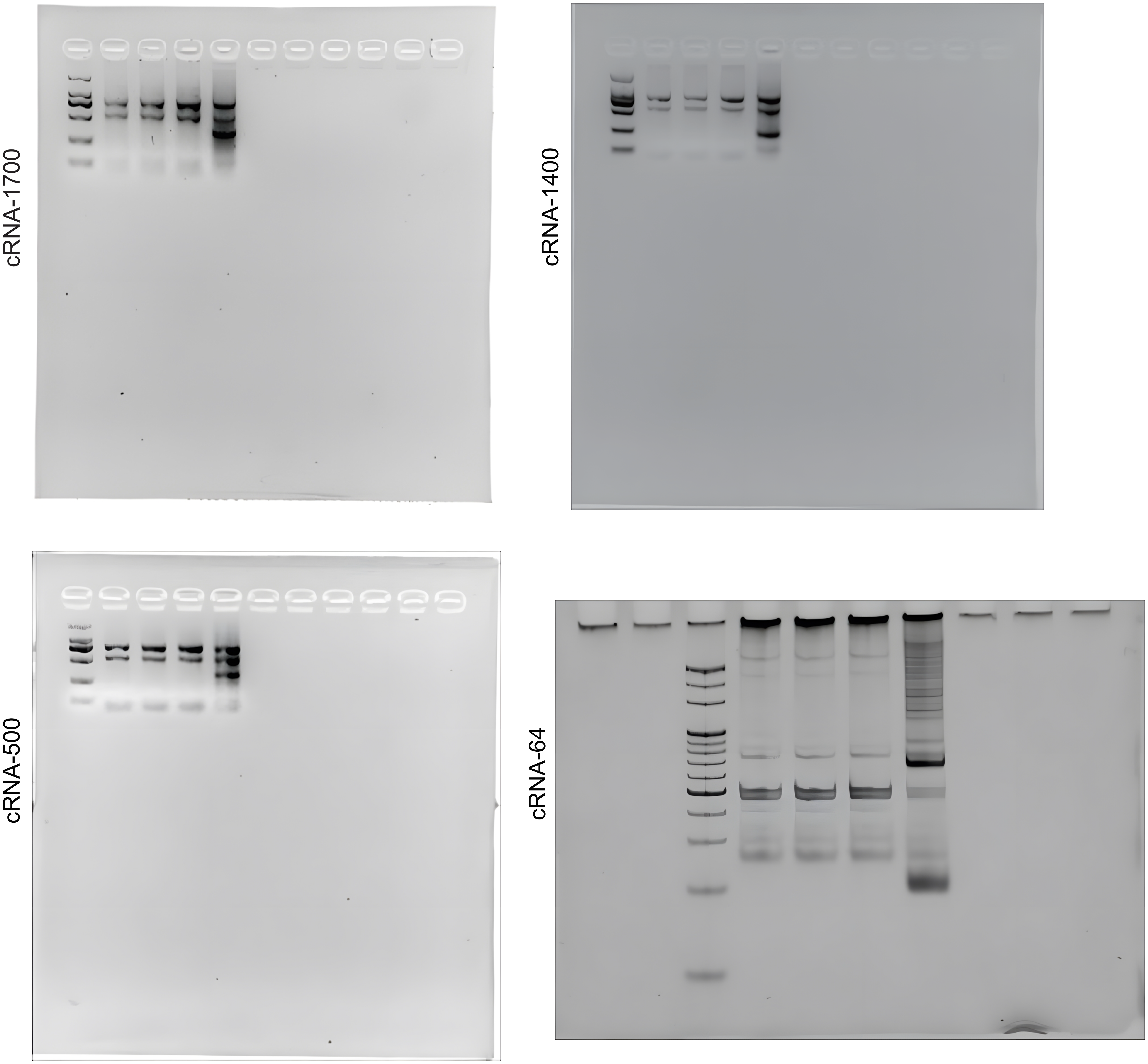

Figure 3A

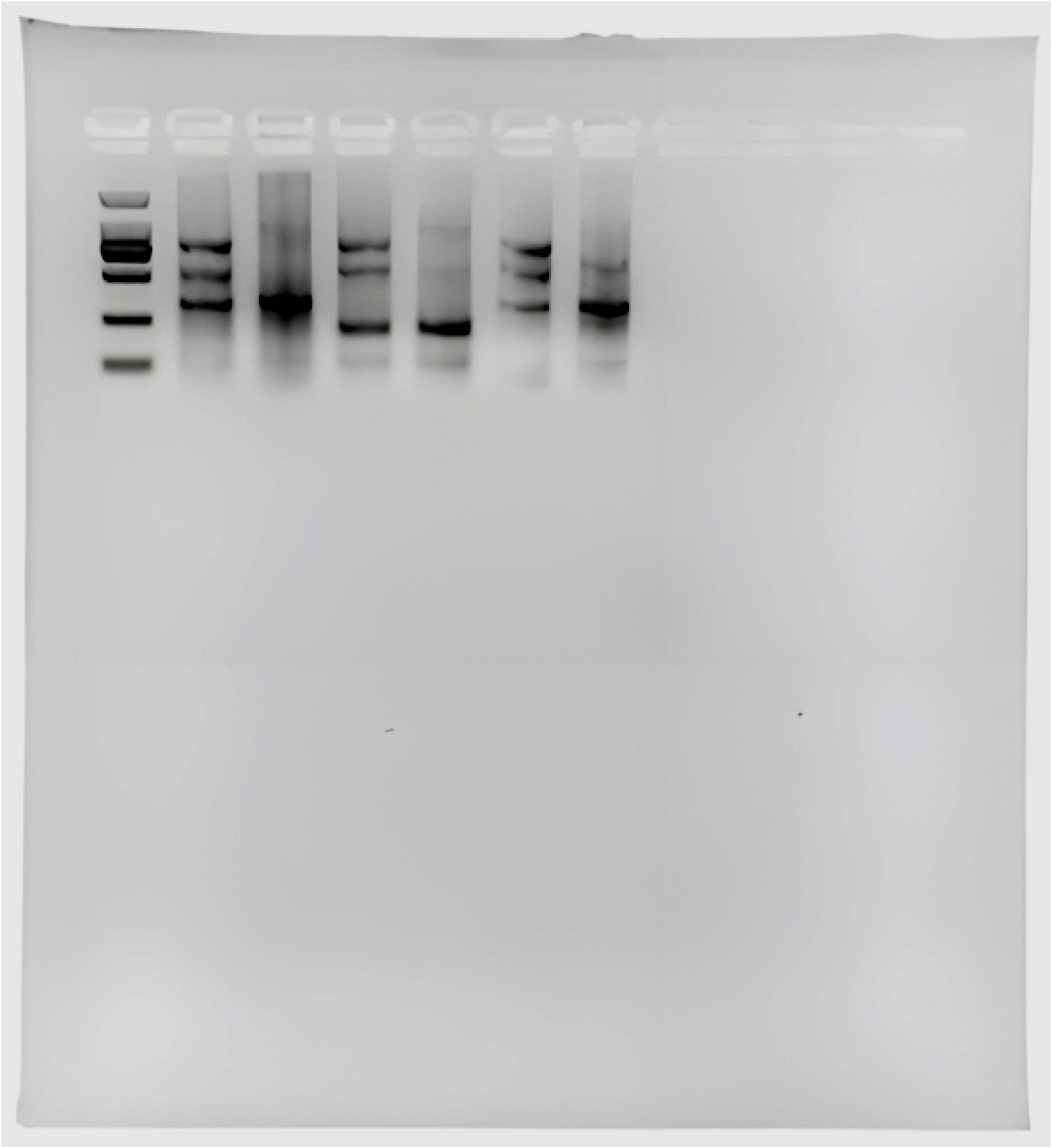

Figure 3B

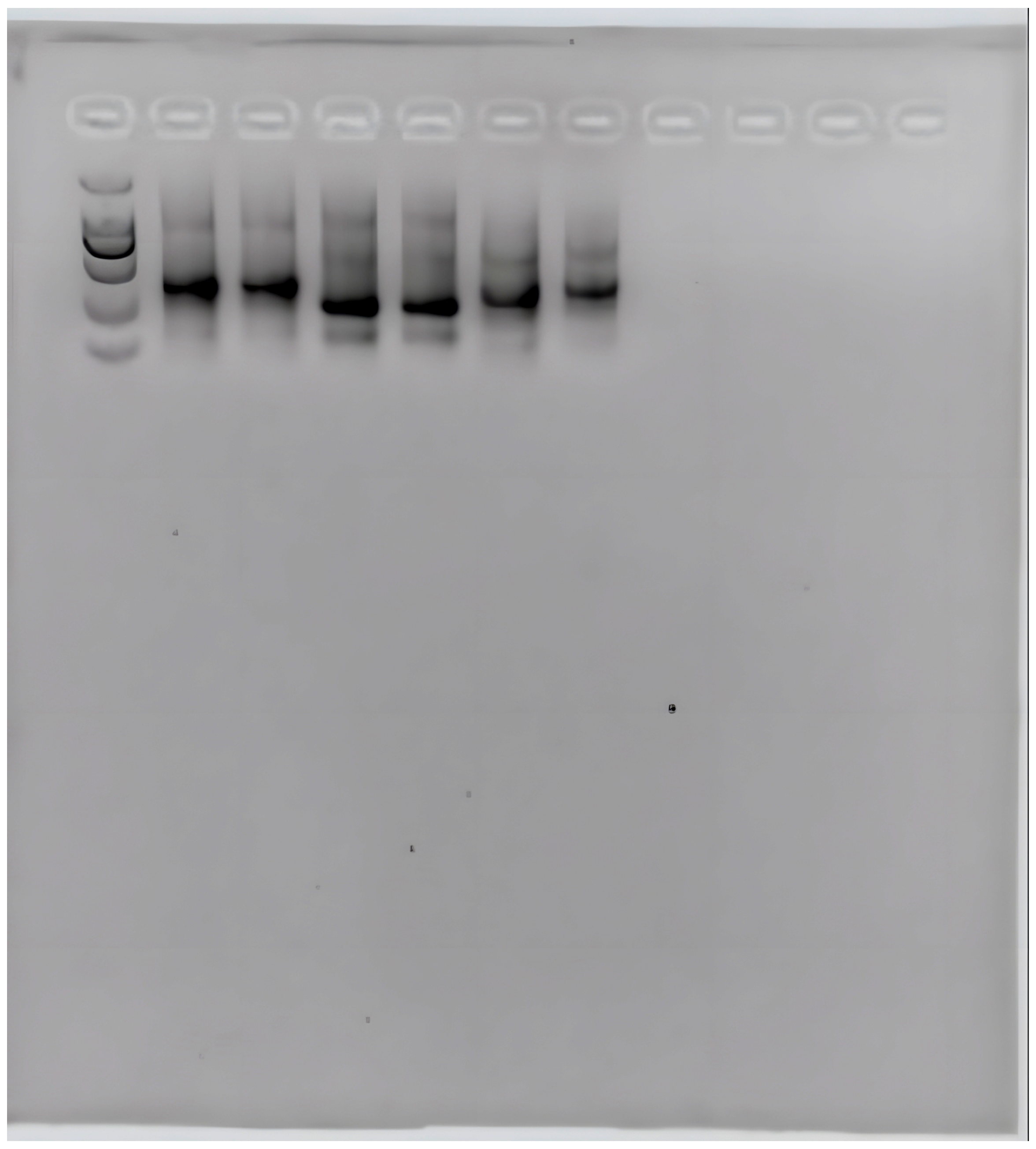

Figure 3C

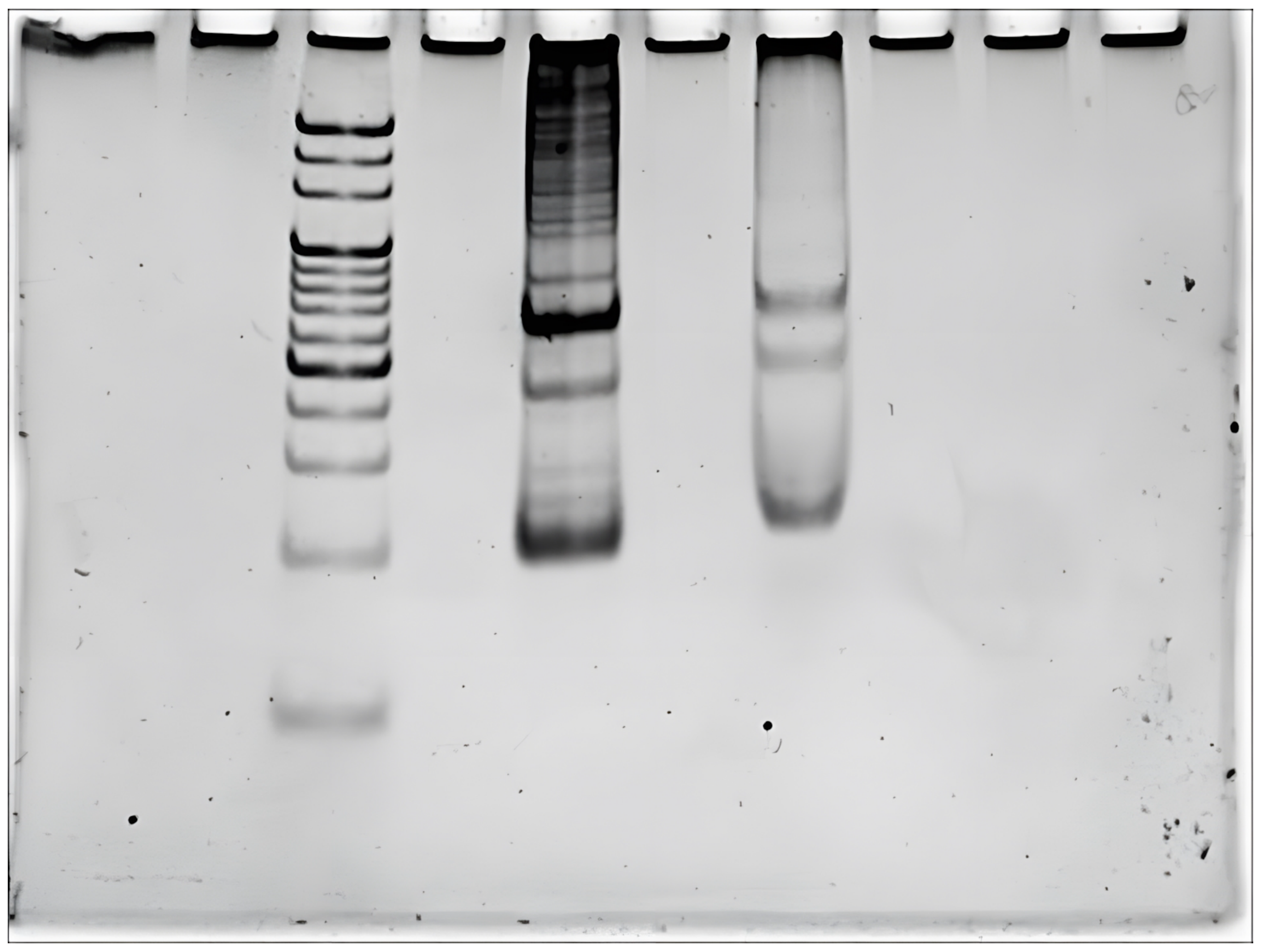

Figure 3C

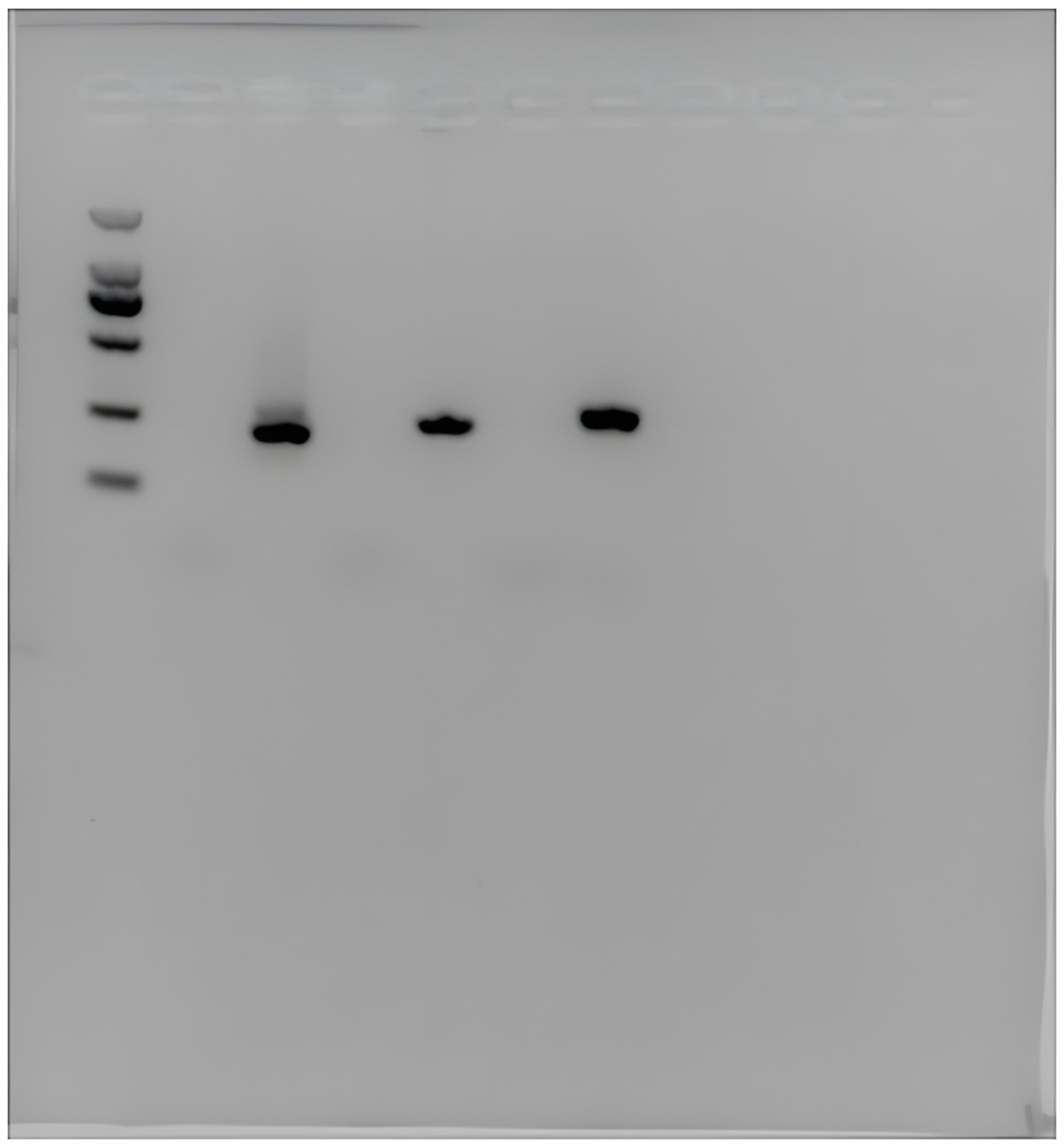

Figure 3D

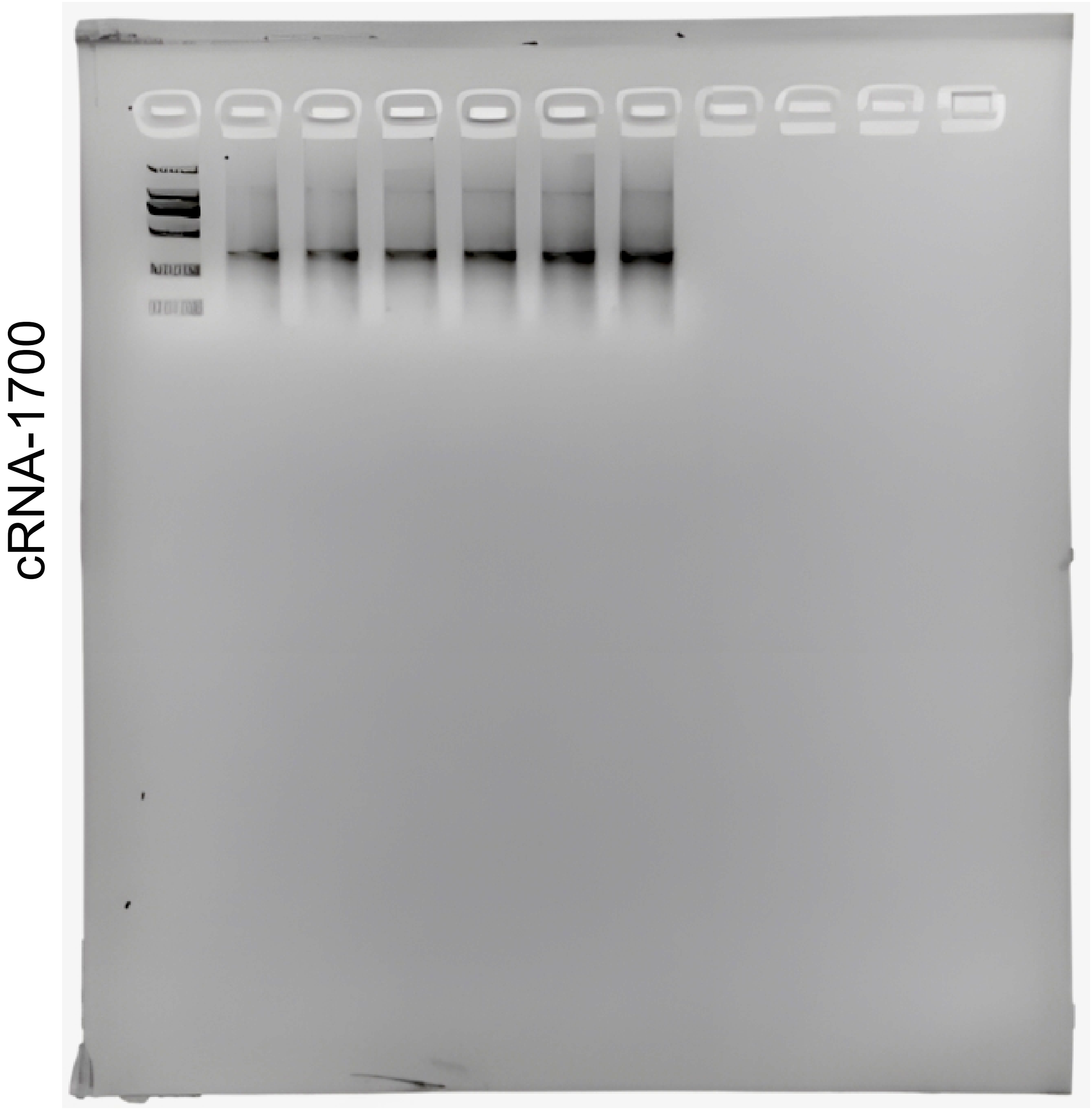

Figure 4A

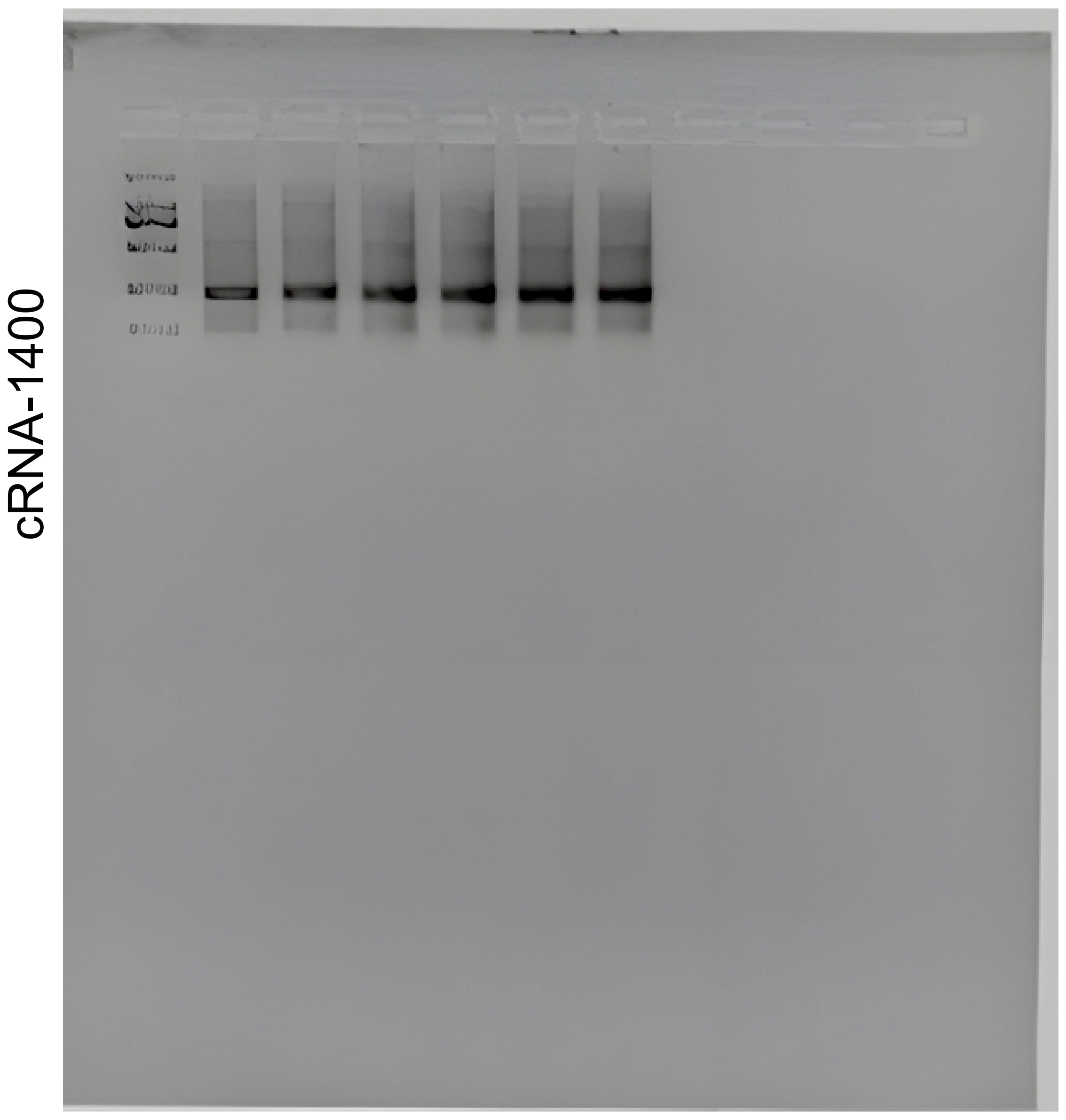

Figure 4A

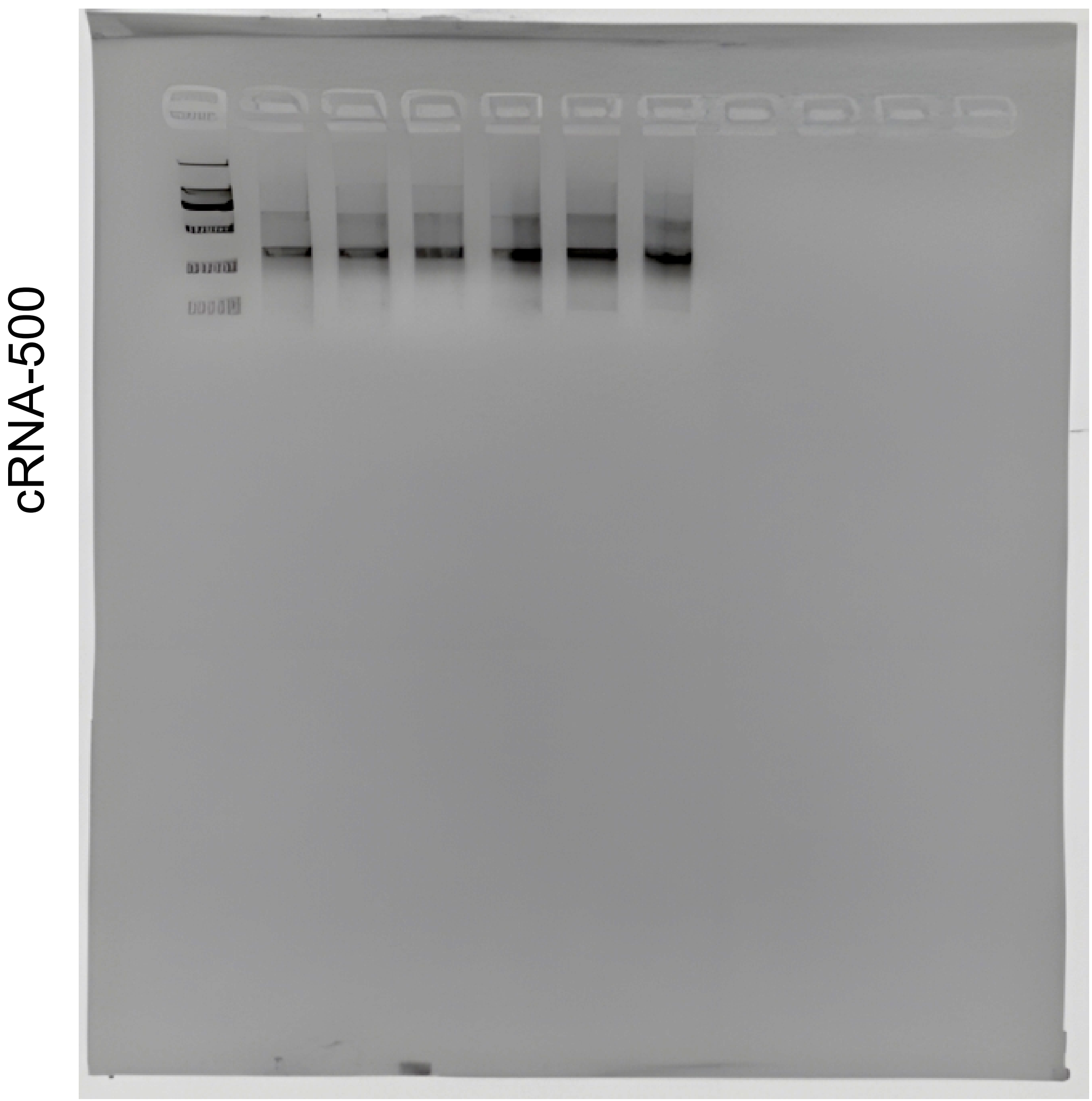

Figure 4A

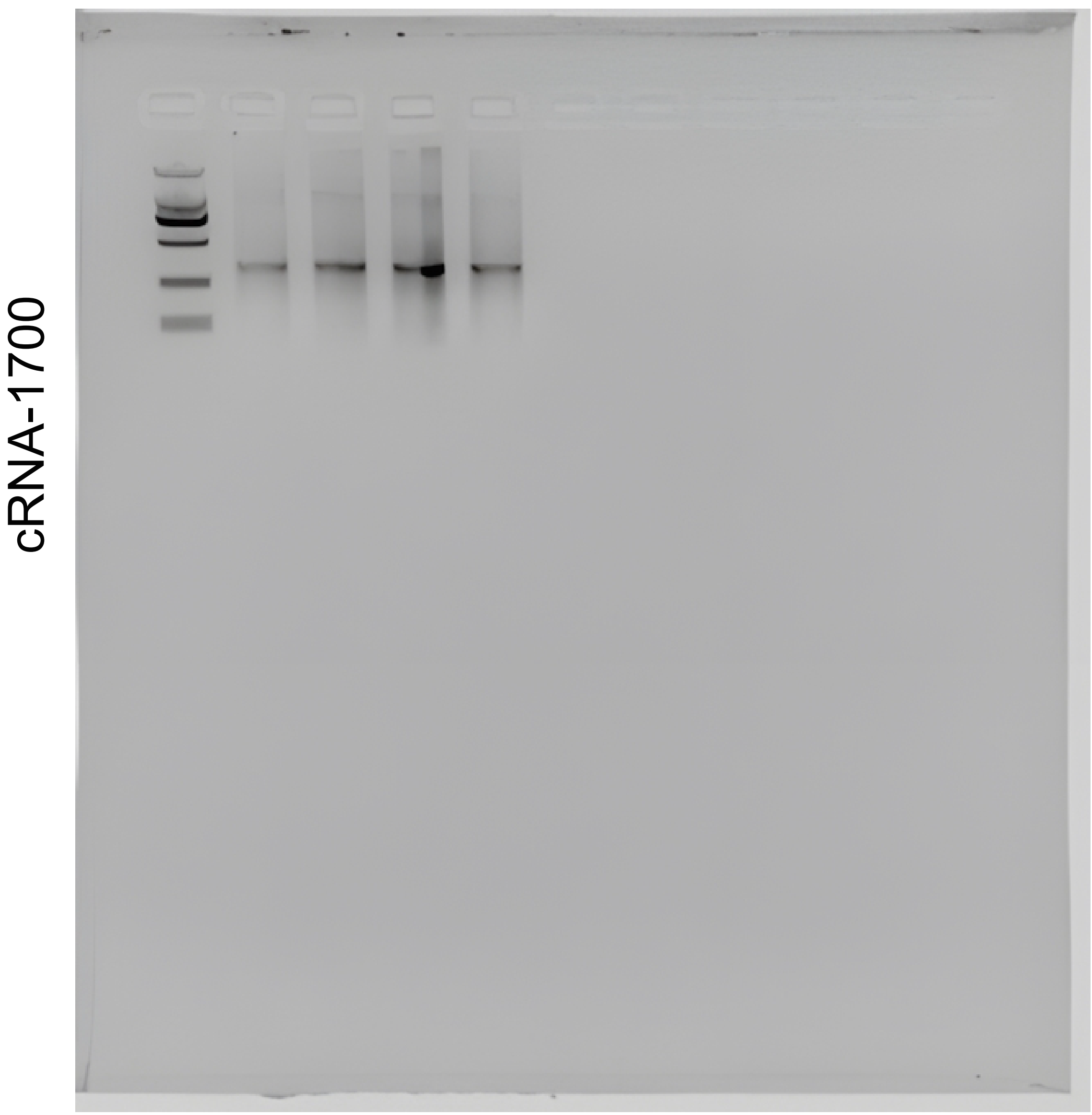

Figure 4B

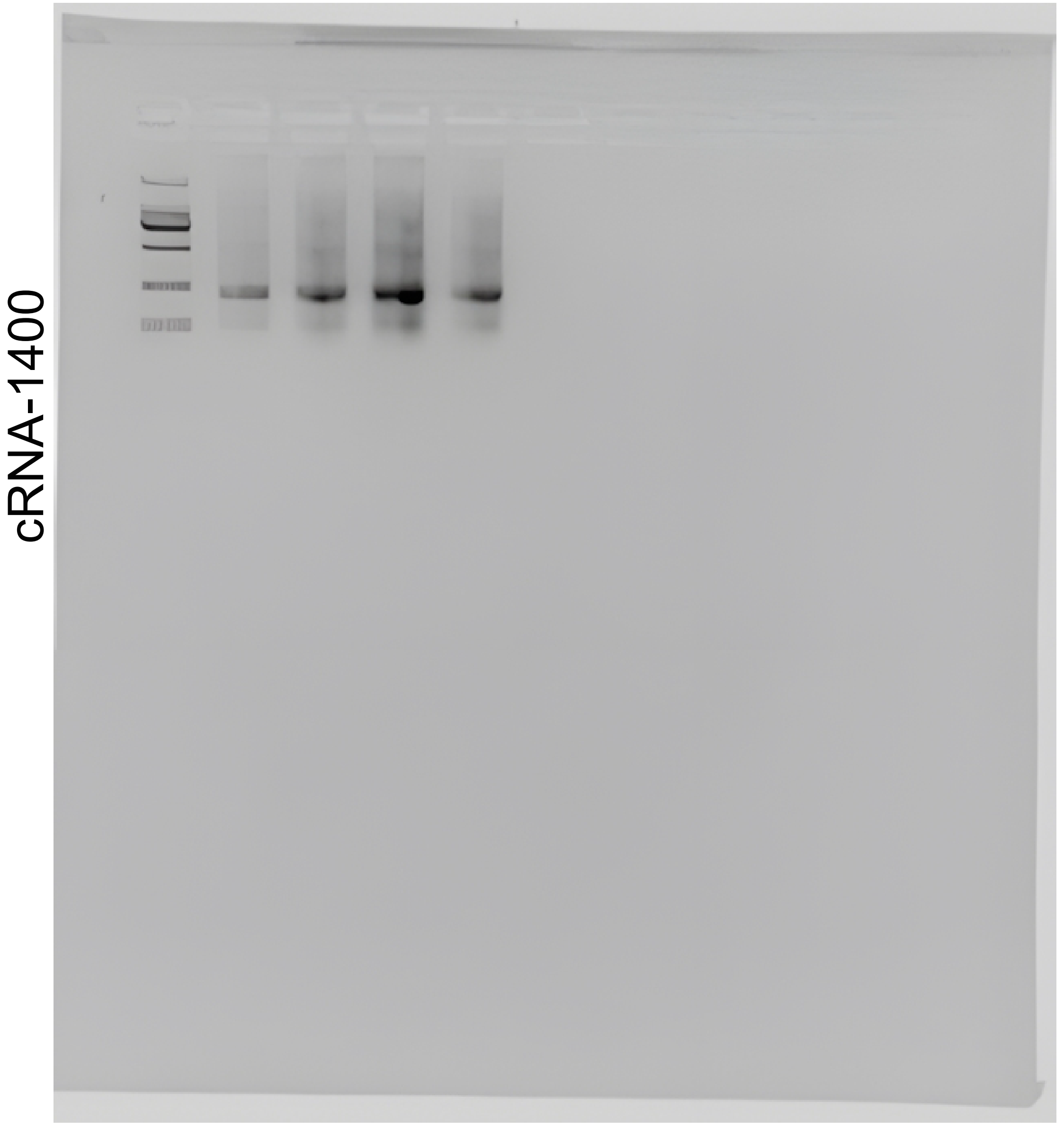

Figure 4B

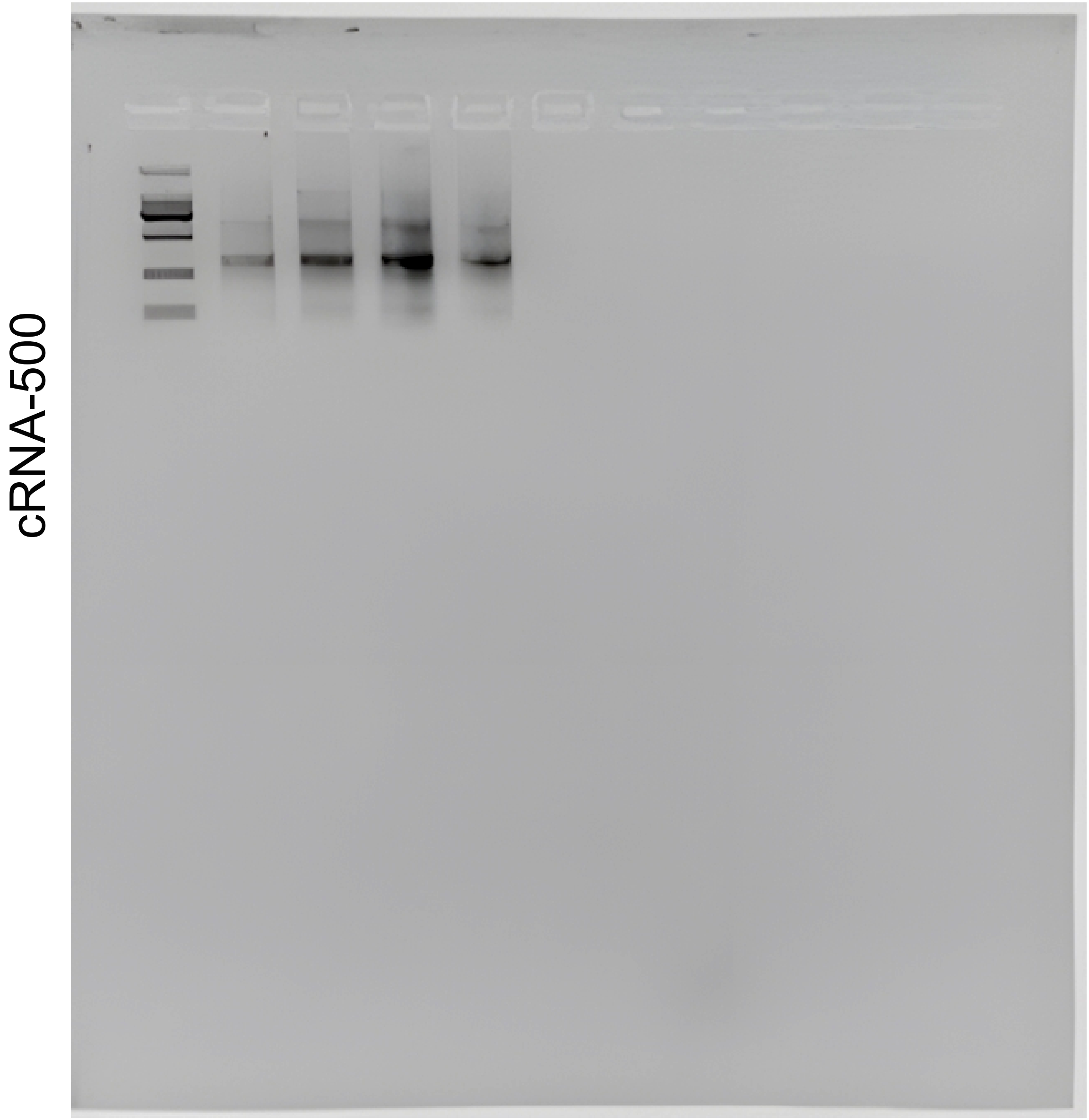

Figure 4B
